# Involvement of the 3’ Untranslated Region in Encapsidation of the Hepatitis C Virus

**DOI:** 10.1371/journal.ppat.1005441

**Published:** 2016-02-11

**Authors:** Guoli Shi, Tomomi Ando, Ryosuke Suzuki, Mami Matsuda, Kenji Nakashima, Masahiko Ito, Tsutomu Omatsu, Mami Oba, Hideharu Ochiai, Takanobu Kato, Tetsuya Mizutani, Tatsuya Sawasaki, Takaji Wakita, Tetsuro Suzuki

**Affiliations:** 1 Department of Infectious Diseases, Hamamatsu University School of Medicine, Shizuoka, Japan; 2 Department of Virology II, National Institute of Infectious Diseases, Tokyo, Japan; 3 Division of Virology, Department of Microbiology and Immunology, Institute of Medical Science, University of Tokyo, Tokyo, Japan; 4 Research and Education center for Prevention of Global Infectious Diseases of Animals, Tokyo University of Agriculture and Technology, Tokyo, Japan; 5 Research Institute of Biosciences, Azabu University, Kanagawa, Japan; 6 Proteo-Science Center, Ehime University, Ehime, Japan; University of California, San Diego, UNITED STATES

## Abstract

Although information regarding morphogenesis of the hepatitis C virus (HCV) is accumulating, the mechanism(s) by which the HCV genome encapsidated remains unknown. In the present study, in cell cultures producing HCV, the molecular ratios of 3’ end- to 5’ end-regions of the viral RNA population in the culture medium were markedly higher than those in the cells, and the ratio was highest in the virion-rich fraction. The interaction of the 3’ untranslated region (UTR) with Core *in vitro* was stronger than that of the interaction of other stable RNA structure elements across the HCV genome. A foreign gene flanked by the 3’ UTR was encapsidated by supplying both viral NS3-NS5B proteins and Core-NS2 in *trans*. Mutations within the conserved stem-loops of the 3’ UTR were observed to dramatically diminish packaging efficiency, suggesting that the conserved apical motifs of the 3´ X region are important for HCV genome packaging. This study provides evidence of selective packaging of the HCV genome into viral particles and identified that the 3’ UTR acts as a *cis*-acting element for encapsidation.

## Author Summary

Although cell culture systems provide a powerful tool for deciphering the life cycle of the hepatitis C virus (HCV), the mechanisms of encapsidation of the viral genome into infectious particles remain to be uncovered. The HCV genome is a positive RNA with one single reading frame flanked by 5’- and 3’ untranslated regions (UTRs). Thus far, there is no direct evidence that HCV employs a packaging-signal dependent- or replication-coupled mechanism of encapsidation of its genome. The possible overlap of RNA sequences that function in RNA replication with those that function in encapsidation may present an obstacle to investigation of the *cis*-elements for RNA packaging. In this study, we characterized the properties of HCV RNAs in a cell culture system by determining their integrity in virus-replicating cells and in culture supernatants, and we found that over-distributed 5’-subgenomes were negatively selected during virus assembly in the cells. Using *trans*-packaging systems with replication defective subgenomes, we identified the 3’UTR as a *cis*-acting element that was sufficient for packaging of not only a HCV subgenome but also a foreign gene into infectious particles. Mutagenesis analyses, together with an *in vitro* binding assay with Core demonstrated that, whereas the best encapsidation occurs with the entire 3’ UTR, the loop sequences of the 3’ X region appear to be essential for encapsidation. Our work opens new perspectives for understanding the molecular mechanisms that regulate the HCV life cycle and potentially paves a way to a new anti-viral therapy.

## Introduction

It is known that positive strand RNA viruses package their genome via replication-coupled processes [1–3]. Nevertheless, for several viruses, viral particle assembly occurs via recognition of particular sequences or structures termed packaging signals that are unique to the viral genes. These signals are more conserved than other parts of the viral genomes and are usually highly structured. However, thus far, a packaging signal has not been identified for any member of the *Flaviviridae* family.

The genome of HCV is a positive strand RNA with a single open reading frame flanked by highly conserved and structured untranslated regions (UTRs) at each end [4]. The 5’ UTR contains determinants for cap-independent translation and *cis*-acting elements for RNA replication. The 3’ UTR contains a short variable region, a poly (U/UC) tract with an average length of 80 nt (nucleotide) and a conserved 98-nt X-tail region (3’ X). Direct interactions of the 3’ UTR and the translation machinery facilitate efficient initiation of subsequent translation [5]. Mutations in the 3’ X region were shown to abort replication [6,7], which illustrates the important role of the 3’ UTR in replication. Both the length and the composition of the poly (U/UC) tract are critical for HCV genome replication [8].

In this study, by using replication-defective *trans*-packaging systems, we identified that the 3’ UTR of the HCV genome acts as a *cis*-acting element for RNA packaging. Within the 3’ UTR, the loop sequences of stem-loop structures appear to be essential for HCV RNA packaging.

## Results

### Molecular ratios of 3’ to 5’ ends of HCV RNA in cells, culture supernatant and density fractions in a HCV cell culture system

Excess amounts of the 5’ end of subgenomic HCV RNA species, which presumably resulted from premature termination of transcription or RNA cleavage in the viral genome, have been detected in the livers and sera of hepatitis C patients [9,10]. To elucidate the properties of HCV RNAs throughout replication and encapsidation, we investigated the distribution of viral RNAs in a HCV cell culture (HCVcc) system. We hypothesized that, if certain viral RNA species are selectively incorporated into virions, then different 3’- to 5’-end molecular ratios of HCV RNA would be observed in virion-rich fractions of the culture supernatant compared to those in the whole cell supernatant and in the cells.

HCV RNAs were determined using Quantitative (q) RT-PCR reactions targeting the 5’ UTR (5’ end) and the NS5B region approaching the 3’ UTR (3’ end) (S1A Fig). The molecular ratios of 3’- to 5’ end were then calculated using RNA copy numbers of NS5B and the 5’ UTR. Firstly, serial dilutions of *in vitro* synthesized HCV RNA of approximately full-genome length, IVT-F (S1B Fig), were analyzed using these two qRT-PCRs. RNA copy numbers of each dilution were determined by targeting the 5’ UTR and the NS5B region (S1C Fig). The ratios of the RNA copy numbers obtained from the two PCR analyses were calculated for each dilution, and their average value of 0.459 was set as a reference ratio (S1D Fig). In the subsequent assays, the “3’:5’-end ratio” was determined by normalizing the ratio of the HCV RNA copy numbers obtained in the two PCR analyses, with the reference ratio.

**Fig 1:**
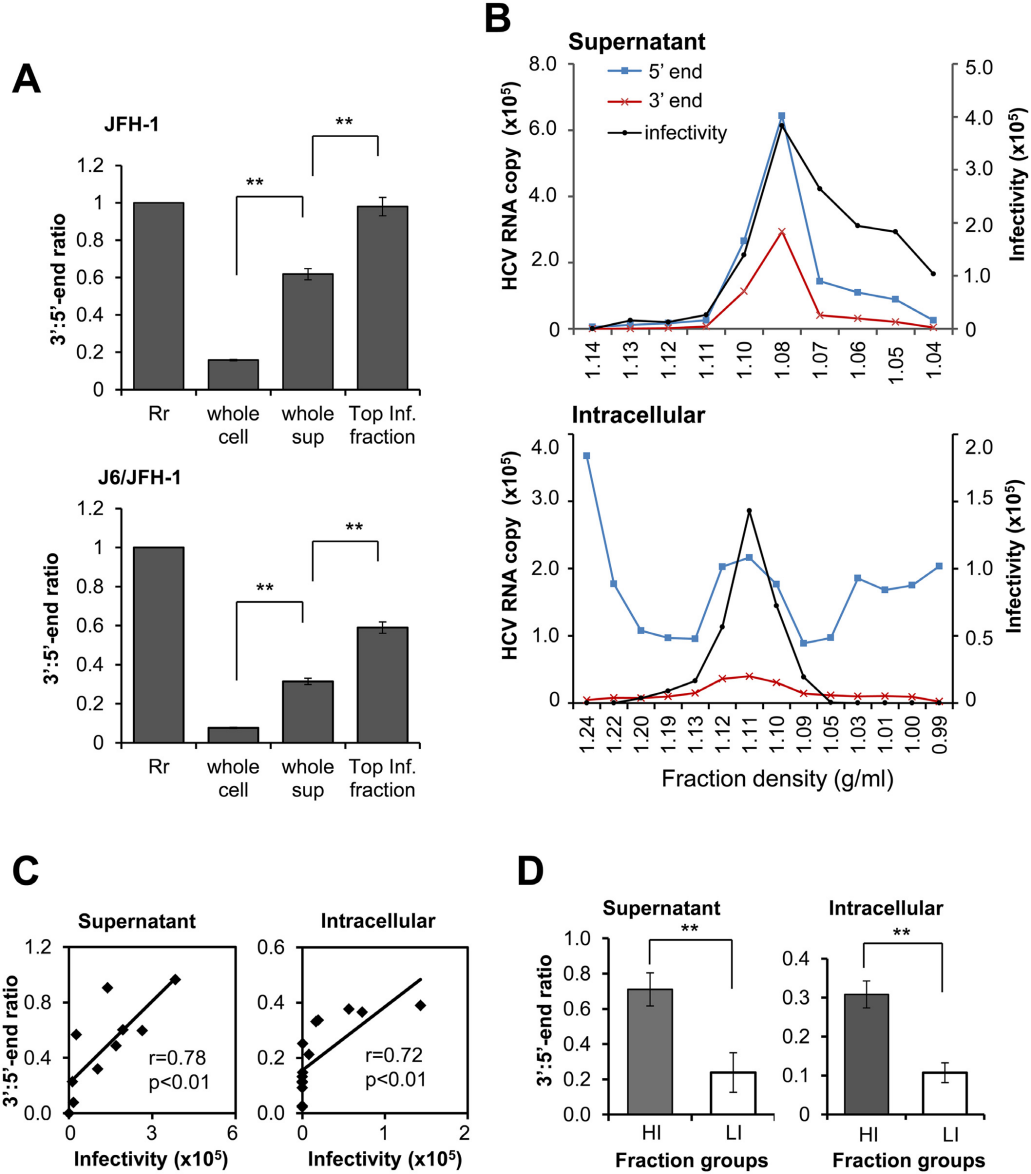
Characteristics of HCV RNAs in infected cells and culture supernatant. (**A**) Normalized 3’:5’-end ratios of HCV RNA from cells (whole cell), supernatants (whole sup) and fractions with the highest infectivity (Top Inf. fraction) of cultures infected with HCVcc JFH-1 or J6/JFH-1. The ratio values calculated from NS5B (3’ end) and 5’ UTR (5’ end) qRT-PCR were normalized by the reference ratio (0.459; S1D Fig). The reference ratio was arbitrarily set to 1 (Rr) and the normalized 3’:5’-end ratios were shown. (**B**) Distribution of HCV RNA (5’ end, 3’ end) in fractions from culture supernatants and lysates of cells infected with HCVcc JFH-1, and the infectivity of each fraction. The y-axis indicates number of HCV RNA copies/ml (left) and infectivity in terms of viral RNA copies per μg of total RNA (right) from cells inoculated with equal aliquots of each fraction. Infectivity was measured by quantification of HCV RNA in the infected cells, 2 days post-infection. Blue, red and black lines represent quantity of 5’ end, 3’ end and infectivity, respectively. (**C**) Correlation of 3’:5’-end ratios with infectivity of the fractions obtained from supernatant and cells following HCVcc (JFH-1) infection as shown in (**B**). Correlations were estimated by way of linear regression and statistical significance was set at *P* = 0.01. (**D**) Comparison of 3’:5’-end ratios of high infectious (HI) and low infectious (LI) fractions. The median value of the infectivity of the fractions was used to split the fractions into HI and LI groups. Fractions derived from JFH-1, as shown in Fig 1B were used. Values are the mean ± SEM (n = 4 for whole cell and whole sup; n = 2 for Top Inf. fraction, n = 5 for supernatant HI and LI fraction groups and n = 7 for intracellular HI and LI fraction groups); ** *P*<0.01, Student’s *t* test.

Total RNAs isolated from cells infected with HCVcc, JFH-1 or J6/JFH-1 (whole cell) as well as their culture supernatant (whole sup), were quantified by using 5’- and 3’-end specific qRT-PCRs. In whole cell preparations, an excess amount of 5’-end HCV RNA was observed compared to 3’-end HCV RNA. The 3’:5’-end ratios in cells infected with JFH-1 and J6/JFH-1 (whole cell) were 0.158 and 0.076, respectively, which were markedly lower than the ratios in the whole sup (for both JFH-1 and J6/JFH-1) (Fig 1A). The supernatants collected from both the HCVcc-infected cells as well as the cell extracts were subjected to density fractionation followed by quantification of the 5’- and 3’-ends of HCV RNA and determination of the infectivity of each fraction (Fig 1B). The distribution of 3’- and 5’-end viral RNA in fractions of the culture supernatant showed a similar pattern, with the highest RNA levels observed in the fraction with the highest infectivity (Fig 1B, upper). As for the cell-derived fractions, the distribution pattern of the 3’-end RNA differed considerably from that of the 5’-end RNA; while the former was mainly detected in infectious fractions, the latter was broadly distributed throughout the fractions tested (Fig 1B, lower). The 3’:5’-end ratios in density fractions with the highest infectivity from the JFH-1 (Fig 1B, upper) and J6/JFH-1 (S2A Fig) cultures were significantly higher than those observed in the whole supernatant (Fig 1A). In addition, 3’:5’-end ratios calculated from the data with normalization shown in Fig 1B correlated positively with infectivity (supernatant; r = 0.78, intracellular; r = 0.72) (Fig 1C and S2B Fig). The fractions were categorized as high or low infectious groups based on the median value of the infectivity of the fractions. The mean value of 3’:5’-end ratios was significantly higher in high infectious fractions (HI) than in low infectious fractions (LI) (Fig 1D). HCV RNA in infected cells was assessed by Northern blotting with a 5’ UTR anti-sense RNA probe. In addition to a major band of viral RNA of around the genome length, considerable signals at a size smaller than 0.5 kb were observed in cells infected with HCVcc (S3 Fig). Combined with the data of the 3’:5’-end ratios in cells, these results suggested that the highly abundant 5’-end containing HCV subgenomes found in the virus-infected cells were negatively selected during the late steps of the viral life cycle.

### Application of a *trans*-packaging system based on a replication-defective subgenomic replicon to identify the *cis*-acting element required for encapsidation

Based on the above findings that the 3’:5’-end ratios of HCV RNA were higher in the culture supernatant, particularly in highly infectious density fractions, relative to those in cells of HCVcc cultures, we deduced that a *cis*-acting sequence, possibly a region containing the 3’ side of the HCV genome, may be involved in encapsidation of the viral RNAs. Although coupling of genome replication and encapsidation has been widely reported among positive-strand RNA viruses, a replication-coupled packaging mechanism for HCV has not been identified to date. Previous studies suggested that HCV potentially employs a virion assembly process which is independent of RNA replication [11], [12]. We thus assessed how HCV RNA replication influences the efficiency of virus assembly by using a *trans*-packaging system, which produces *trans*-complemented HCV particles (HCVtcp), as previously described [13].

**Fig 2:**
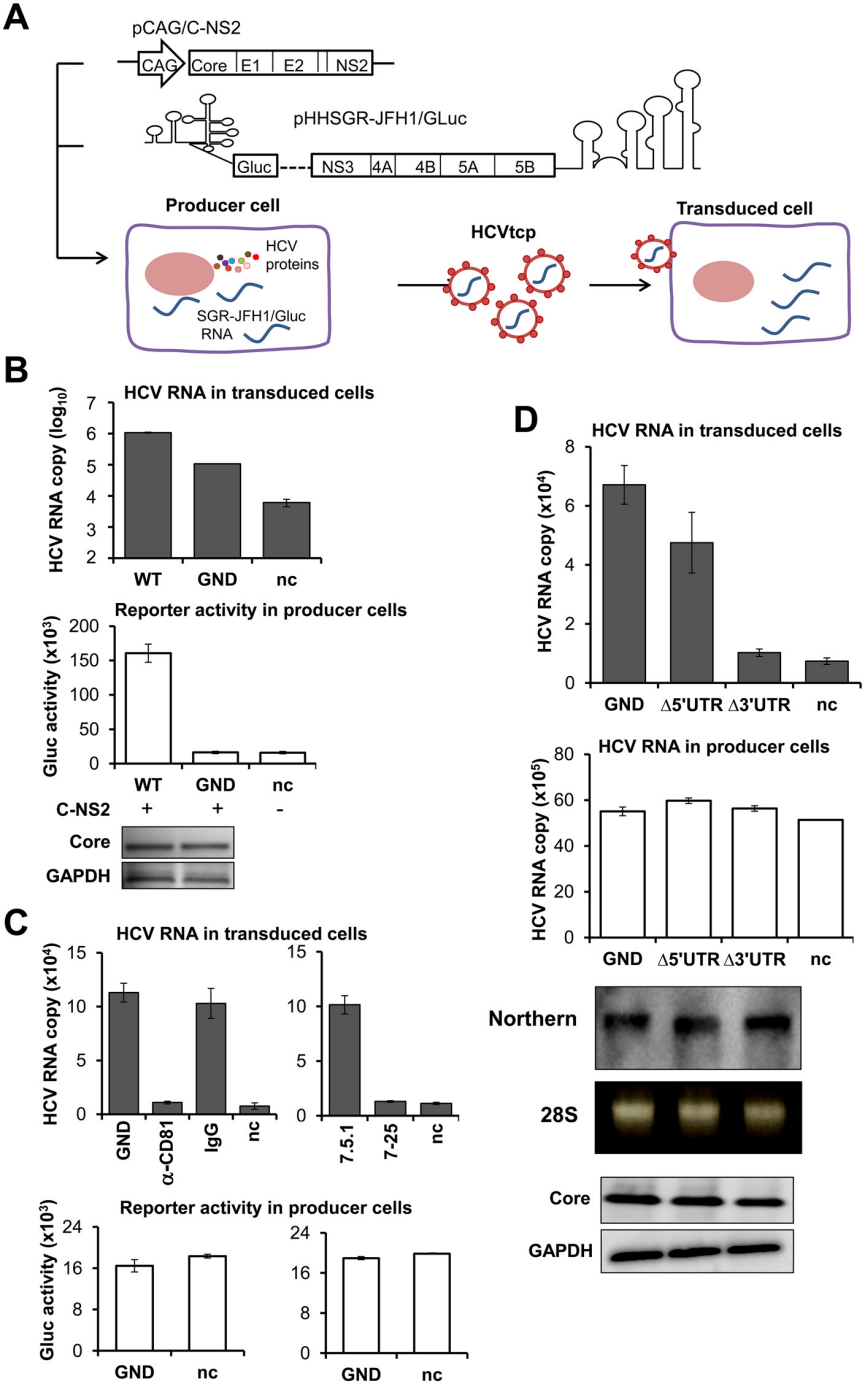
*Trans*-packaging system based on a replication-defective subgenomic replicon. (**A**) Schematic representation of the HCV *trans*-packaging system. (**B**) Production of HCVtcp from replication-competent or replication-defective subgenomic replicon (WT or GND, respectively). Transfection with an empty vector pCAG-Neo and pHHSGR-JFH1/Gluc/GND was used to determine the background control (nc), unless described elsewhere. HCVtcp production was determined by quantification of the viral RNA in the transduced cells at 12 hr post-inoculation. The lower panel showed Gaussia luciferase (Gluc) activity released from producer cells. Core expression in producer cells was assessed by immunoblotting. (C) Blocking of HCVtcp entry by anti-CD81 antibody (left), and inoculation of Huh7-25 cells (right). Huh7.5.1 cells were pre-incubated with 20 μg/ml of anti-CD81 antibody (α-CD81) or mouse IgG (IgG) for 1 hr, followed by inoculation with GND HCVtcp. HCV RNA levels in the transduced cells (upper) and Gluc activity released from producer cells (lower) are shown. (**D**) Deletion of UTR impaired production of HCVtcp. Upper graph: Production of HCVtcp from GND and UTR deletion mutants. HCV RNA level in the transduced cells (upper graph) and producer cells (middle graph) were determined by qRT-PCR targeting NS5B region as shown in S1 Fig. Northern blot analysis of HCV RNA in Huh7.5.1 cells transfected with GND, Δ5’ UTR or Δ3’ UTR constructs (lower graph), Huh 7.5.1 cells were transfected with the mutant constructs and subjected to RNA extraction 72 hr post-transfection. 10 μg of total RNA was loaded to formaldehyde denaturing agarose gel electrophoresis and followed by Northern hybridization; a DIG-labeled RNA probe targeting to NS5B was used. 28S rRNA was used to demonstrate equal loading. Comparable Core expression in the producer cells was determined by western blotting. (**B, C, D**) Results of HCV RNA in transduced and producer cells, and reporter activity in producer cells represent the means of three independent experiments ± SEM. HCV RNA copies are indicated as numbers per μg of total RNA for each assay, and Gluc activities are indicated as RLU per μl.

To produce HCVtcp, Huh7.5.1 cells were co-transfected with pHH-based plasmids expressing a replicative (pHHSGR-JFH1/Gluc; WT) or non-replicative (pHHSGR-JFH1/Gluc/GND; GND) JFH-1 subgenome and a plasmid pCAG/C-NS2 encoding Core, E1, E2, p7 and NS2 proteins. HCVtcp was inoculated into naïve Huh7.5.1 cells and transduced HCV RNA was determined (Fig 2A). Although transduced HCV RNA in the cells inoculated with culture supernatants from GND- and Core-NS2-expressing cells was about 10-fold lower than that from WT-expressing cells, the transduced RNA levels were sufficient for detection (Fig 2B). Transduced RNAs were detected with pre-treatment of the inoculum with benzonase, which proved that the transduced RNAs were introduced by nuclease-resistance structures (S4 Fig). Anti-CD81 antibody clearly blocked RNA transduction via inoculation of culture supernatants from GND- and Core-NS2-expressing cells. In addition, Huh7-25 cells that are not susceptible to HCV entry due to a lack of CD81 expression [14] were not transduced, confirming the production of HCVtcp with a replication-defective JFH-1 genome (Fig 2C).

To test for pHH-based constructs for which it might be feasible to later analyze by mutational scanning to identify *cis*-elements of the HCV genome packaging, we removed the 5’- or the 3’ UTR of the GND subgenome. It was noted that production of HCVtcp dramatically decreased, close to the background level, when the 3’ UTR of pHHSGR-JFH1/Gluc/GND was deleted. Only a limited influence on HCVtcp production was observed by deletion of the 5’ UTR (Fig 2D upper). Deletion of either UTR did not impair RNA stability (Fig 2D, Northern blotting). Comparable expression of the mutant subgenomes and HCV structural protein was detected in the producer cells (Fig 2D, middle and lower graphs).

The combined results strongly suggested that replication of HCV RNA is important for efficient virus production but is not indispensable for viral assembly. Deletion of the UTR regions in the pHH-based construct did not essentially affect RNA stability. Thus, this *trans*-packaging system based on a replication-defective subgenome, which is sensitive enough to detect relatively low levels of HCVtcp, can be applied to investigate *cis*-acting signals for encapsidation of HCV RNA, independent of RNA replication.

### The 3’ UTR functioned as a *cis*-acting signal for RNA encapsidation

**Fig 3:**
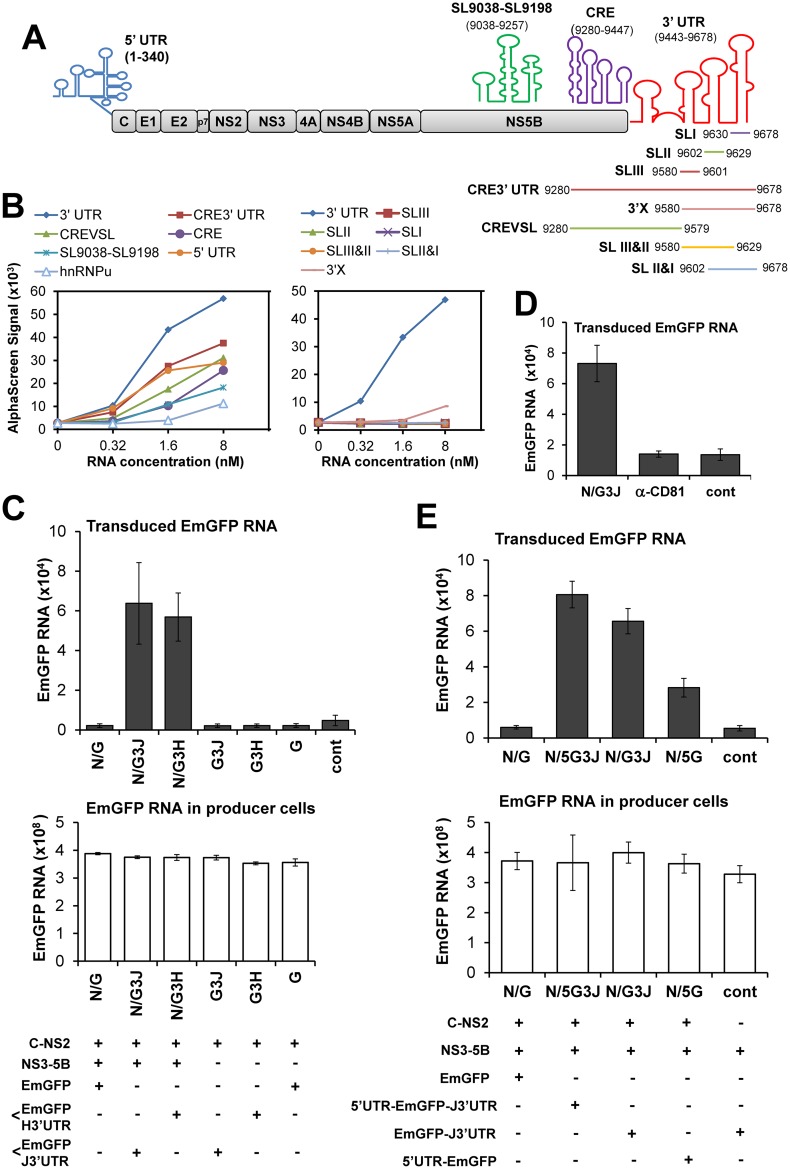
Entire 3’ UTR is required for Core-binding to produce HCVtcp. (**A**) Schematic representation of HCV genome and the regions used for the Core-RNA interaction assay. Stem-loops and colored lines depict *in vitro* synthesized and folded RNA fragments used. (**B**) *In vitro* interactions of RNA fragments with Core determined by AlphaScreen. Results for comparison among structure clusters across HCV genome (left) and among 3’ UTR and the fragments within the region (right) were obtained from two independent assays. (**C**) *Trans*-packaging of EmGFP RNA into HCV particles indicated by transduced RNA level in the inoculated cells (upper). Transduction of EmGFP RNA was determined at 12 hr post-inoculation. EmGFP RNA level in the co-transfected producer cells (lower) is shown. N: pCAG/NS3-5B, G: p/EmGFP, G3H: p/EmGFP-H3’UTR, encoding EmGFP followed with 3’ UTR of H77. G3J: p/EmGFP-J3’UTR, encoding EmGFP followed with 3’ UTR of JFH-1. cont; control with cells co-transfected with a pCAG-Neo empty vector, p/EmGFP-J3’UTR and pCAG/NS3-5B. (**D**) Entry of HCVtcp was blocked by anti-CD81 antibody (α-CD81), carried out as described in Fig 3C. Data were present as mean ± SEM, n = 4. (**E**) Comparison of *tran*-packaging of EmGFP RNAs, directed by 3’- or 5’ UTR. 5G3J: p/5’UTR-EmGFP-J3’UTR, encoding EmGFP flanked by 5’ UTR and 3’ UTR of JFH-1. 5G: p/5’UTR-EmGFP, addition of 5’ UTR at upstream of EmGFP. (**C, D, E**) Results shown represent the means of three independent experiments ± SEM. RNA copies are indicated as numbers per μg of total RNA.

To investigate molecular mechanism(s) of encapsidation of HCV RNA, we determined the interactions of Core with a variety of HCV RNA fragments that have conserved sequences among HCV isolates and that can potentially fold into highly ordered stem-loop structures (Fig 3A and S5 Fig). The interaction strength between in vitro synthesized Core tagged with an N-terminal FLAG and a series of biotinylated HCV RNAs was assessed by using AlphaScreen [15]. Core interacted with all of the RNA fragments tested. Notably, the highest assay signal was observed with the entire 3’ UTR (3’ UTR), compared to the signals obtained with the 5’ UTR, nt 9038–9257 (SL9038-SL9198), the 3’-163 nt of NS5B (CRE), CRE plus the 3’ UTR (CRE3’UTR), the RNA deleting 3’ X tail of CRE3’UTR (CREVSL) or the 268 nt-RNA fragment derived from the cellular hnRNPU gene (Fig 3A and 3B, left). CRE is known to form a long distance kissing-loop structure with SLII of the 3’ UTR, which is crucial for viral RNA replication [8]. Addition of CRE to the 3’ UTR (CRE3’UTR) resulted in reduction of the Core-3’ UTR interaction. Core interaction with RNA elements within the 3’ UTR was then further assessed (Fig 3A and 3B, right). Neither single- (SLI, SLII, and SLIII) nor double- (SLI&II, SLII&III) stem-loop structures, nor the 3’X region of the 3’ UTR exhibited efficient interactions with Core, compared to interaction of the entire 3’ UTR. Thus, it is likely that Core preferably binds to a pocket or to surface RNA structures composed of virtually the entire 3’ UTR. Indeed, deletion of the entire 3’ UTR from pHHSGR-JFH1/Gluc/GND caused a dramatic decrease in HCVtcp production (Fig 2D).

These findings encouraged us to test whether the 3’ UTR of HCV is sufficient to allow packaging of a foreign RNA sequence into HCVtcp. The HCV 3’ UTR sequence derived from JFH-1 (genotype 2a) or H77c (genotype 1a) isolates was inserted into a reporter plasmid, p/EmGFP, downstream of the coding region of Emerald Green Fluorescent Protein (EmGFP), yielding p/EmGFP-3’UTR (S6A Fig). Huh7.5.1 cells were transfected with p/EmGFP or p/EmGFP-3’UTR together with pCAG/C-NS2 and a plasmid encoding the NS3-5B polyprotein, pCAG/NS3-5B (S6A Fig). We found that the EmGFP-3’ UTR but not the EmGFP sequence was packaged when C-NS2 and NS3-NS5B polyproteins were simultaneously supplied (Fig 3C, upper). HCVtcp packaged with the EmGFP-3’ UTR was not produced in the absence of NS3-NS5B expression, demonstrating the involvement of NS proteins in HCV assembly. The 3’ UTR sequences derived from JFH-1 and H77c supported a comparative level of encapsidation of EmGFP RNA into HCVtcp (Fig 3C, N/G3J VS N/G3H). Cell entry of the EmGFP-3’ UTR-packaged HCVtcp was blocked by anti-CD81 antibody (Fig 3D). EmGFP, Core and NS5A expression, as well as EmGFP RNA levels in producer cells, were confirmed (S6B and S6C Fig and Fig 3C, lower). The transduced RNA levels were sufficient for detection with pre-treatment of the inoculum with benzonase (S6D Fig). Using this strategy, we tested whether the 5’ UTR of HCV supported encapsidation of EmGFP RNA. Although the 5’ UTR sequence appeared to support RNA encapsidation to some extent, the efficiency was significantly lower than that of the 3’ UTR (Fig 3E). A moderate increase in transduced EmGFP RNA was detected when it was flanked with both the 5’ UTR and the 3’ UTR, compared to the version followed with the 3’ UTR alone (Fig 3E). Competitive binding assays demonstrated that the 5’ UTR fragment did not compete with the 3’ UTR for Core-binding (S6E Fig). When considered together with the HCVtcp production shown in Fig 2D and the *in vitro* binding data shown in Fig 3B, these findings suggested that, although both UTRs may be involved in HCV genome packaging, the packaging function of the 3’ UTR is higher compared to that of the 5’ UTR.

**Fig 4:**
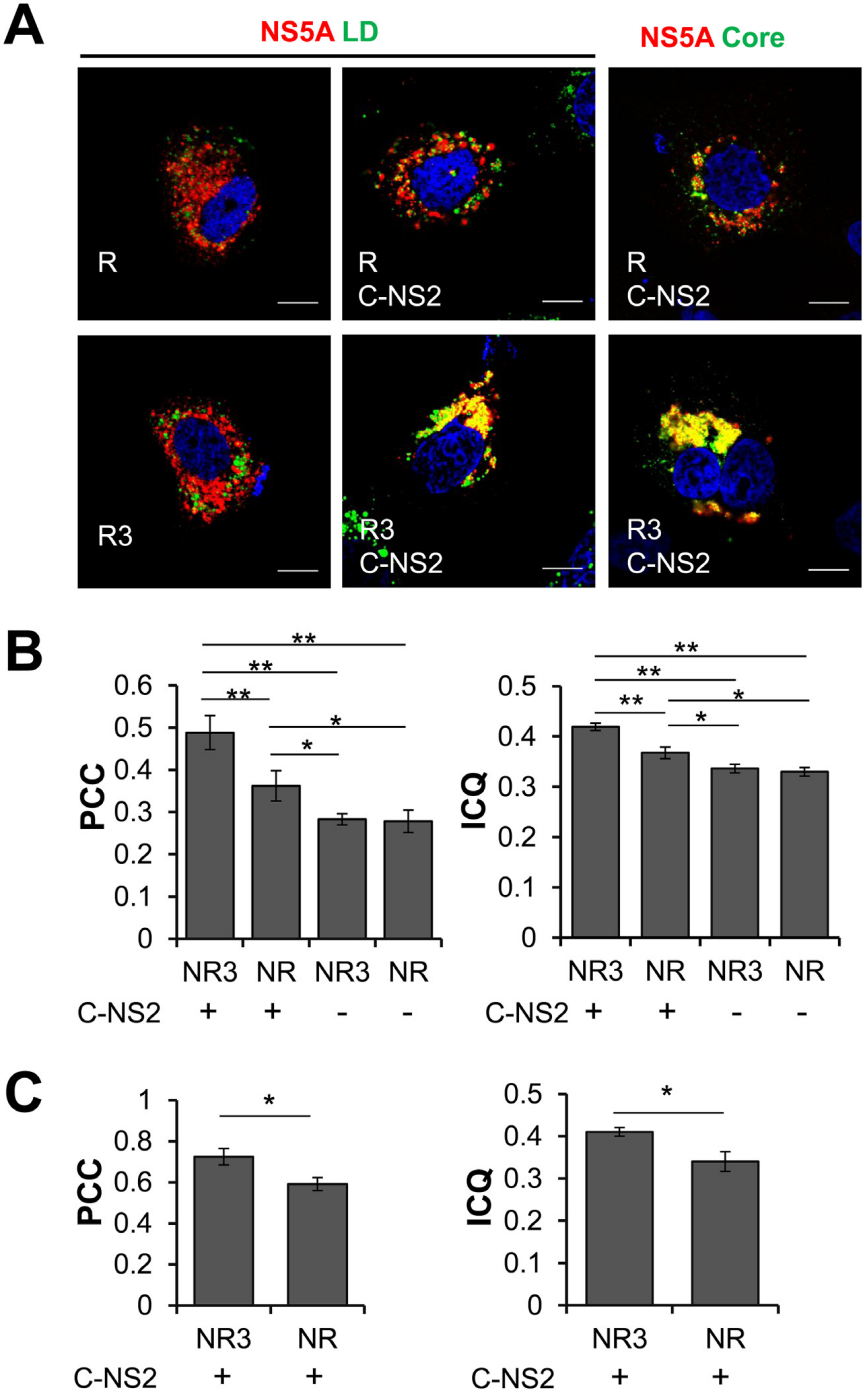
(A) Subcellular localization of NS5A (red), counter stained with LD (green in left and middle panels) or with Core (green in right panels). Huh7.5.1 cells were co-transfected with pCAG/NS3-5B, pRluc (R) or pRluc-3’UTR (R3), together with pCAG/C-NS2 (C-NS2) (middle and right panels) or an empty vector (left panel), and were immunostained for NS5A, Core and LD at 48 hr post-transfection. Scale bars represent 20 μm. Co-localization between NS5A and LD (**B**) or NS5A and Core (**C**) was assessed by Pearson’s correlation coefficient (PCC) and intensity correlation quotient (ICQ) analyses. For each group, co-localization was analyzed in 30 cells. N: pCAG/NS3-5B, R: pRluc, encoding Rluc; R3: pRluc-3’UTR, encoding Rluc followed with 3’ UTR of JFH-1. Data were present as mean ± SEM, n = 30. * *P*<0.05, ** *P*<0.01, Student’s *t* test.

To further investigate the microenvironment of foreign RNA (*Renilla luciferase* (Rluc) RNA in this setting) packaging into HCVtcp, we assessed the localization of HCV proteins in producer cells using confocal laser scanning microscopy (Fig 4). Huh7.5.1 cells were co-tranfected with pCAG/NS3-5B, pRluc (R) or pRluc-3’UTR (R3), together with pCAG/C-NS2 (C-NS2) or an empty vector, and immunostained for NS5A and lipid droplets (LD) or NS5A and Core after 48 hr. The co-localization of NS5A with LD or Core was further analyzed by quantifying the Pearson’s correlation coefficient (PCC) [16] and intensity correlation quotient (ICQ) [17]. The degree of co-localization of NS5A and LD was significantly increased when Rluc-3’ UTR and Core-NS2 were co-expressed (R3, C-NS2), compared to expression of Rluc (R) alone, Rluc plus Core-NS2 (R, C-NS2), or Rluc-3’ UTR (R3) (Fig 4A, left and middle, and Fig 4B). NS5A-Core co-localization was also significantly increased in cells expressing Rluc-3’ UTR with Core-NS2, compared to cells expressing Rluc with Core-NS2 (Fig 4A, right and Fig 4C). These results suggested that the 3’ UTR sequence facilitates the interaction between Core and NS5A at or around LD in HCVtcp-producing cells.

These combined findings demonstrated that the 3’ UTR was more capable of interacting with Core and directing packaging of foreign RNA into HCV particles than the 5’ UTR. The 3’ UTR functioned as a *cis*-acting signal for efficient encapsidation and, not only Core-NS2, but also NS3-NS5B polyproteins were involved in *trans*-complementing viral assembly as a means to encapsidate foreign RNA in combination with the HCV 3’ UTR.

### The entire 3’ UTR of HCV is involved in efficient packaging

**Fig 5:**
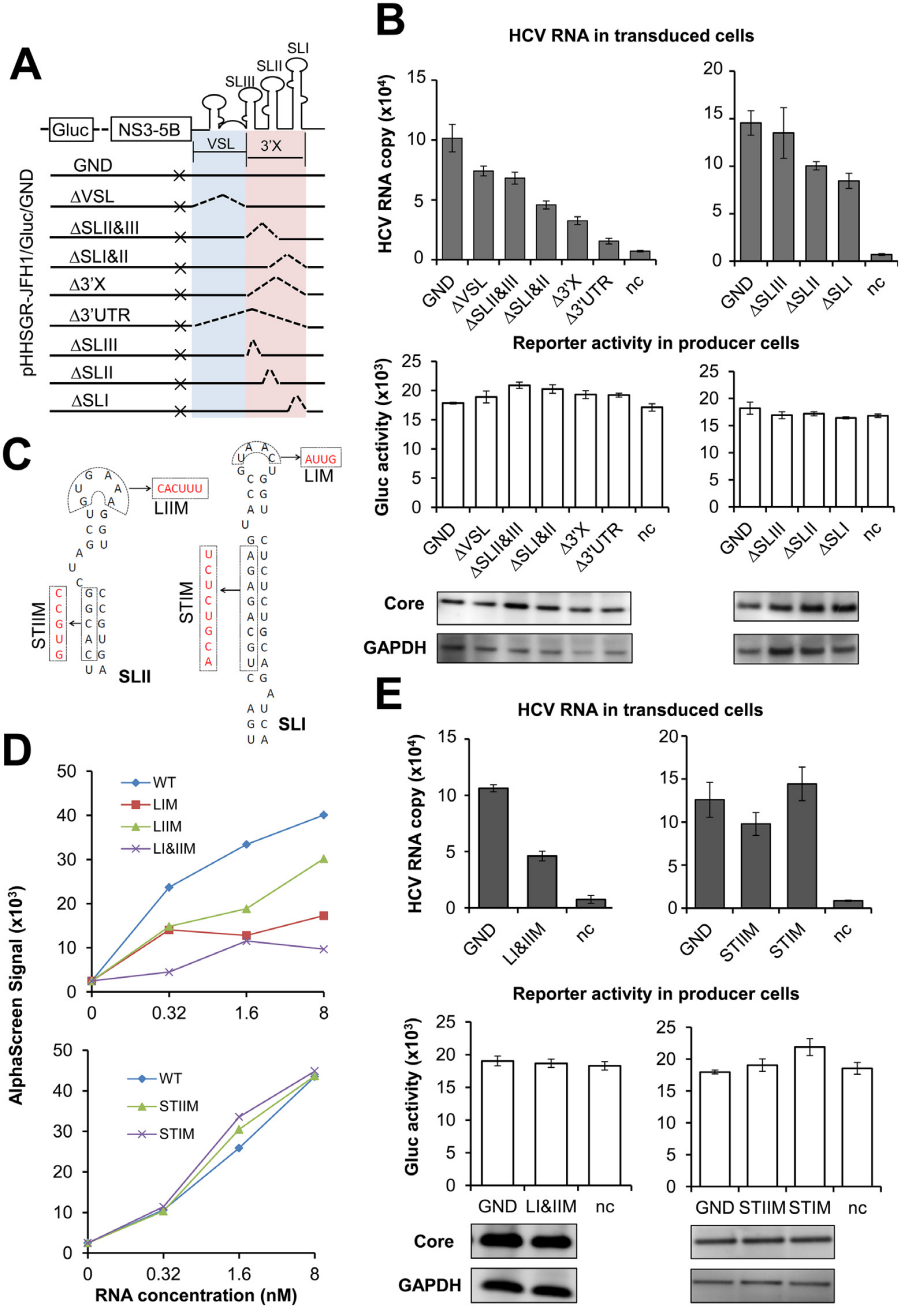
Effect of mutations in 3’ UTR on Core-binding and HCVtcp production. (**A**) Schematic representation of designed mutants in replication-defective subgenomic replicon SGR-JFH1/Gluc/GND. Colored shadows and dashed lines were used to depict the deletion boundaries. (**B**) Production of HCVtcp by using the mutant subgenomic replicons. HCVtcp production (upper) and expression of subgenome (Gluc) and Core in the producer cells (middle and lower) were shown. (**C**) Predicted structures of SLI and II of 3’ UTR are depicted along with the substitution mutations introduced. The resultant mutants were named STIM, STIIM, LIM, LIIM and LI&IIM. (**D**) The interactions of Core with 3’ UTR mutants shown in (**C**). (**E**) Production of HCVtcp by replication-defective subgenomic replicons with 3’ UTR mutations shown in (**C**). HCVtcp production (upper), expression of subgenome and Core in the producer cells (middle and lower) are shown. HCVtcp production was determined as in Fig 2. Results shown represent the mean of three independent experiments ± SEM. HCV RNA copies are indicated as numbers per μg of total RNA for each assay, and Gluc activities are indicated as RLU per μl. VSL: variable region and poly (U/UC) tract, 3’X: the 3’ X tail, ×: GND mutants in NS5B.

To further address the role of the 3’ UTR in packaging of HCV RNA, we introduced a series of deletion mutations into the 3’ UTR of the non-replicative subgenome pHHSGR-JFH1/Gluc/GND (GND), and examined the effects of these mutations on the production of HCVtcp. The resultant mutants were ΔSLI, ΔSLII and ΔSLIII, in each of which one stem-loop of the 3’ X region was deleted; ΔVSL, in which the variable region and the poly (U/UC) tract were deleted; and ΔSLII&SLIII, ΔSLI&SLII, and Δ3’X, in each of which two or three stem-loops of the 3’ X region were deleted (Fig 5A). Comparable levels of mutant and GND RNAs in the producer cells demonstrated comparable stability of the RNAs expressed (S7 Fig). All of the mutants showed equivalent expression of Gluc to GND (Fig 5B, middle), which also indicated that a similar level of RNA was expressed under the pol I promoter. The expression level of Core in the producer cells was also comparable (Fig 5B, lower). As described above, deletion of the whole 3’ UTR (Δ3’UTR) markedly impaired HCVtcp production by 85% compared to that of GND. The mutants Δ3’X, ΔSLI&II, ΔSLII&III and ΔVSL reduced HCVtcp production by 68%, 55%, 33% and 27%, respectively. Among mutants with deletions of single stem-loop structures, deletion of either SLI or SLII, but not of SLIII, reduced HCVtcp production to some extent (Fig 5B, upper). These results, together with the data of RNA-Core binding (Fig 4B), suggested that the entire 3’ UTR of the HCV genome is important for efficient encapsidation. The 3’ X region, particularly SLI and SLII in this region, was indispensable for efficient encapsidation, while the variable region and poly (U/UC) stretch were also possibly involved in packaging.

To verify the importance of SLI and SLII in these processed, effects of mutations in these regions (Fig 5C) on Core binding and HCVtcp production were determined. The interaction of the 3’ UTR with Core *in vitro* was significantly reduced when nucleotides in the loop regions of SLI and SLII were replaced with their paired counterparts (LIM, LIIM, LI&IIM) (Fig 5D, upper). Conversely, the interactions of the 3’ UTR with Core were maintained when substitution mutations were introduced into the stem regions of SLI and SLII (STIM and STIIM) (Fig 5D, lower). Accordingly, mutations in the loops but not in the stems of SLI and SLII led to a marked decrease in HCVtcp production (Fig 5E). When LI&IIM mutations were engineered into the full-length JFH-1 genome, both genome replication and infectious virus production were markedly impaired as expected. Notably, a greater level of reduction of virus production than of genome replication was detected (S8 Fig). On day 3 post-transfection with pHH-JFH1 (WT) and pHH-JFH1-X-LM (X-LM; JFH-1 with LI&IIM mutations), the viral RNA level was around 5-fold lower in the cells replicating the mutant JFH-1 than in the cells with wild-type. In contrast, the mutant JFH-1 produced a 25-fold lower level of infectious particles than the wild type. This finding demonstrated that the apical sequences in stem-loops I and II are not only involved in HCV genome replication but also affect step(s) in virion assembly, and this result is consistent with the findings regarding Core-interaction and HCVtcp production.

Based on the collected data, we propose a model whereby encapsidation of the HCV genome is potentially triggered by direct contact of Core with loop regions in the 3’ X region of the 3’ UTR, while other RNA structures of the 3’ terminus act as brace backbones to support this interaction and/or nucleocapsid formation.

## Discussion

Evidence regarding the virion assembly of HCV has accumulated over the past years; however, the detailed mechanisms responsible for incorporation of viral genomes into progeny virions remain largely unclear.

A large proportion of 5’ end subgenomic HCV RNA with heterogeneous termination sites has been reported in the livers of hepatitis C patients [9,10], consistent with a phenomenon found in viral-replicating cells. The progressive increase in 3’:5’-end ratios of HCV RNA in highly infectious density fractions and in whole-culture supernatant relative to viral replicating cells indicated selective packaging of integrated genomes into infectious particles. In the light of these results, we speculate that HCV may use a packaging-signal-dependent encapsidation pathway. Known packaging signals for RNA viruses are usually located in highly structured and conserved regions. The most structured and conserved sequences of HCV are the untranslated regions at either end. The findings that HCV RNAs containing a 3’-end side were enriched through particle assembly encouraged us to determine whether the 3’-end region of the HCV genome contains *cis*-packaging elements. Formation of a pseudoknot structure through a kissing interaction between the 3’ X region of the 3’ UTR and CRE within the NS5B region is essential for viral RNA replication [18]. Taking into account that the signals required for encapsidation might directly overlap with those required for replication, adoption of an approach by which the packaging process can be uncoupled from replication/translation is required for investigation of the process of encapsidation. Here we demonstrated that production of virions with both replicative and non-replicative subgenomes was possible in Huh7.5.1 cells. Notably, either 3’- or 5’-UTR deletion impaired HCVtcp production, but neither of these deletions perturbed RNA stability. Deletion of the 3’ UTR resulted in a decrease in transduced HCV RNA to the background level, while deletion of the 5’ UTR caused a minor reduction. It may be possible that the 5’ UTR is also somehow involved in efficient packaging of the HCV genome.

In general, initiation of selective encapsidation of a viral genome requires recognition of a packaging signal by the nucleocapsid protein. Several conserved RNA structures across the HCV genome (the 5’ UTR, SL9038-SL9198, CRE in the NS5B-coding sequence, and the 3’ UTR) were tested for *in vitro* interaction with Core. Interestingly, we found that the interaction of CRE in the NS5B-coding sequence with Core turned out not to be as strong as the interaction of the 3’ UTR with Core. When the 3’ UTR was linked to CRE (CRE3’UTR), the strength of its interaction with Core was lower than that of the 3’ UTR alone. It was likely that access of Core to the 3’ UTR was implicated in the conformational switch of RNA-RNA interactions such as the kissing-loop structure formed via the interaction between CRE and the 3’ X region. Previous observations suggested that structural rearrangements within the 3’ end region of the HCV genome are important for the regulation of switching between different steps of the HCV lifecycle [19–21].

Furthermore, the HCVtcp system was modified by introducing a reporter gene cassette (EmGFP-3’ UTR) for encapsidation. Based on the results of the two HCVtcp systems and the RNA-Core interaction assay, we concluded that the 3’ UTR of the HCV genome functions as a *cis*-acting element for RNA packaging. The available evidence suggests that the packaging signal for the HCV genome is not contained within the 5’ UTR [22]. Our experimental approaches indicated that the 5’ UTR potentially supports packaging of foreign RNA. However, it does so significantly less efficiently than the 3’ UTR, which was consistent with our observation of impairment of HCVtcp production by deletion of the 3’- or the 5’ UTR. The packaging efficiency of the 3’ UTR was not compromised by the 5’ UTR when these two RNA structures were simultaneously supplied in separate cassettes.

It appears that the SLI and SLII regions of the 3’ UTR play a key role in HCV encapsidation. Furthermore, mutations in the loops of SLI or SLII, which were predicted not to affect RNA secondary structures, led to strong negative effects on both Core-binding and *trans*-packaging efficiency. Nevertheless, mutations in the stem regions did not influence Core-binding or HCVtcp production. The predicted structures (by RNAstructure software) of STIM and STIIM showed that, even when the substitutions in the stems dramatically changed the RNA structures, there were no major changes in the loop area. Thus, the stem mutants still contained the loop motifs of the wild-type RNA (S9 Fig), which possibly explained the unchanged interaction with Core. Based on the combined data, we deduced that the entire 3’ UTR is needed for efficient packaging, while the apical sequences of SLI and SLII are crucial for this process. When the same mutations of apical sequences were engineered into full-length JFH-1, greater reduction of virus production than that of genome replication was detected, which supported the findings that these sequences are also involved in virion formation independent of replication. Therefore, in HCV, sequence motifs located in the loops of multiple stem-loop RNA structures were crucial for encapsidation, similar to packaging signals of hepatitis B virus and alphaviruses [23,24].

In the setting with EmGFP-3’UTR as a gene cassette for HCVtcp production, it is of interest that co-expression of NS3-NS5B together with Core-NS2 was a prerequisite for packaging of the reporter gene. Presumably, in the HCVtcp system used, the nonstructural proteins did not function as replication machinery but rather contributed to creating the subcellular environment required for virion assembly. In addition to viral structural proteins and p7, all NS proteins contribute to the production of infectious HCV particles [25]. NS2 and p7 are crucial for virus assembly and release [26–31] and can be *trans*-complemented[32]. NS3, NS4A, NS4B, NS5A, and NS5B are indispensable for HCV genome replication and virus assembly [33–36]. Although NS4B and NS5A can be *trans*-complemented for replication [31,37], it has not been determined whether NS3-NS5B can be *trans*-complemented for virion production. Here, for the first time, we demonstrated that all HCV proteins could be supplied in *trans* for virion packaging in a replication-defective HCVtcp system. Impaired *trans*-packing of virions with mutations in domain III of NS5A indicated direct involvement of this domain in encapsidation. The 3’ UTR of the HCV genome has been found to activate an IKK-α-dependent pathway that induces lipogenic genes and enhances Core-associated LD formation to facilitate viral assembly [38]. We observed that association of NS5A with LD and Core was enhanced by co-expression of the 3’ UTR. It is likely that, in addition to its direct role as a packaging signal, the 3’ UTR induces the host cell environment required for facilitation of HCV morphogenesis. Functional coupling of RNA packaging and replication have been reported for several positive strand RNA viruses [1–3]. One may hypothesize that a replication-coupled packaging mechanism might permit efficient access of the nucleocapsid protein so that it can interact with progeny HCV RNA. However, this coupling mechanism may result in competition of Core and the replicase for RNA binding. Thus, temporal recruitment of Core to LDs avoids such competition [39]. It was proposed that the newly synthesized HCV genome RNA, coupled with NS5A, is released from the replication complex-containing membrane vesicles, and recruited to the surface of LDs or their associated membranes [33,35,40,41] where nucleocapsids are assembled.

In this study, we acquired comprehensive knowledge regarding HCV RNA associated with infectious particles and identified the packaging signal of HCV virus encapsidation. The findings discussed here will help to decipher the complicated process of the early phases of virion assembly of HCV. Our work provides a rational framework for refining the molecular mechanisms regulating the HCV lifecycle and potentially enables determination of mechanisms that govern other related positive strand RNA viruses.

## Materials and Methods

### Preparation of HCV RNA with approximate full genome length

The *in vitro* synthesized viral genomic RNA was prepared with a MEGAscript T7 kit (Life technologies), using a linearized HCV cDNA clone as the template. The genome-size RNA was purified from agarose gel after formaldehyde denaturing agarose gel electrophoresis of the synthesized RNA. Uniformity of the IVT-F RNA was assessed by Agilent 2100 Bioanlyzer in combination with the Agilent RNA 6000 Nano Kit.

### Quantitative RT-PCR

Quantification of HCV RNA was performed by qRT-PCRs using TaqMan EZ RT-RNA Core Reagents (Applied Biosystems). HCV RNA quantifications were done by targeting either 5’ UTR or NS5B, in two (q) RT-PCR sets with respective RNA standards. The primers and probes used for quantifying the 5’ UTR of HCV have been described previously [42]. To set up the quantification of HCV RNA targeting the 3’ end of the genome, the 3’-end sequence (nt 8001–9678) of JFH-1 isolate, including partial NS5B and 3’ UTR was subjected to screen the best primers and probes by IDT Scitools RealTime PCR (Integrated DNA Techonologies). Initially, two probes in the NS5B region and one probe in the 3’ UTR region were estimated. The working efficiencies of these probes were compared and the one with best efficiency was picked for quantification of HCV RNA in name of 3’ end qRT-PCR. Thus, the primer/probe set located at NS5B (nt 8057–8196), a forward primer 5'-CAA ACA CCA ATT CCC ACA ACC -3' and reverse primer 5'-TCA TAG AGG GCC ATT TTC TCG -3’, and the probe 5'-/56-FAM/AC CAG CTC G/ZEN/C CTC ATC GTT TAC C/3IABkFQ/-3', were used. The assay for quantification of HCV RNA at 5’ UTR and NS5B was performed as follows: for each sample, RNA was mixed with 3 mM of MnOAc, 10 mM of each dNTP, 10 μM of forward and reverse primer, 10 μM of TaqMan probe, 2.5 U of rTh polymerase, 0.5 U of Amp Erase UNG, in the TaqMan EZ buffer scale to 15 μl with double distilled water. PCR run program: starting with reverse transcription at 50°C for 1 min, 60°C for 50 min and 95°C for 5 min, then followed by 50 cycles of target amplification, denaturation at 94°C for 15 s, annealing at 55°C for 10 s and extension at 72°C for 1 min. A primer/probe set for qPCR targeting EmGFP gene was selected from validated Assays-on-Demand products (RIKAKEN).

Total cellular RNA was extracted with ReliaPrep RNA purification kit (Promega) and treated with TURBO DNase (Life technologies), followed by cleaning up with a Nucleospin RNA clean-up kit (TaKaRa Bio). RNA from supernatant or density fractions was extracted with a SepaGene RNA extraction kit (Edia Japan) according to manufacturer’s instructions.

### Production of HCVtcp and infection

HCVtcp was generated by cotransfection with pCAG/C-NS2 and pHHSGR-JFH1/Gluc essentially as previously described [13]. To produce HCVtcp carrying EmGFP RNA, cells were cotransfected with pCAG/C-NS2, pCAG/NS3-NS5B and either p/EmGFP, p/EmGFP-J3UTR, p/EmGFP-H3UTR, p/5’UTR-EmGFP-J3’UTR or p/5’UTR-EmGFP using TransIT-LT1 (Mirus Bio). At 72 hr post-transfection, cultured media and cells were harvested and assessed for infectivity by inoculating naïve Huh7.5.1 cells and expression of viral proteins by Western blotting, respectively (see S1 Text for details). Culture medium containing HCVtcp was filtered through a 0.45 μm Sterivex filter unit (Millipore) and 20 μl of it was used to measure Gluc activity. The remaining filtered medium was used to inoculate Naïve Huh7.5.1 cells maintained at 37°C with 5% CO_2_. After 3 hr inoculation, cells were washed with PBS and cultured 12 hr with fresh growth medium for RNA extraction.

### Northern blotting

Total RNAs of transfected Huh7.5.1 cells were isolated with Tri-reagent (Sigma-Aldrich), at 72 hr post-transfection. The isolated RNAs were treated with TURBO DNase (Life technologies), followed by cleaning up with a Nucleospin RNA clean-up kit (TaKaRa Bio). The resulted RNAs were then analyzed by electrophoresis in a 1.5% agarose-2.2 M formaldehyde gel, followed by Northern hybridization by using a DIG Northern starter kit (Roche) according to the manufacturer’s instructions. DIG-labeled anti-sense RNA probe complementary to the positive strand of NS5B (nt 7951–8476) was used to detect HCV RNA.

### Amplified luminescent proximity homogeneous assay (AlphaScreen)

Biotinylated RNA fragments were synthesized using a MEGAscript T7 kit (Life technologies) or provided by Sigma Aldrich. The size and stability of RNA fragments synthesized with MEGAscript kit were checked by agarose gel electrophoresis. RNA fragments purchased from Sigma Aldrich were provided in 0.05 μmole scale, HPLC grade purity. FLAG-tagged HCV Core and its mutants were synthesized using a WEPRO *in vitro* translation kit (CellFree Sciences) using pEU/core/wt as a template.

The RNA-Core interaction assays were carried out in a final reaction volume of 25 μl by adding each assay component to the following final concentrations: 0.32 nM, 1.6 nM, or 8 nM of biotinylated RNA, 20 nM of *in vitro* synthesized Core protein, 0.1% BSA (w/v), 40 U RNase inhibitor, 20 μg/ml anti-FLAGm2 acceptor beads, 20 μg/ml streptavidin donor beads, 2.5 μl of 10x AlphaScreen assay buffer (PerkinElmer), followed by 90 min of incubation at 25°C and then subjected for determination of interactions by AlphaScreen signals (photon counts at 630 nm/s). AlphaScreen signals were detected on an EnSpire plate reader (PerkinElmer).

### Preparation of fractionated samples from HCV-infected cell cultures

The culture supernatant of Huh7.5.1 cells infected with HCVcc was concentrated by ultrafiltration with a centrifugal filter device (Amicon), and filtered with a 0.22 μm Sterivex filter unit (Millipore). To release intracellular virus, cell pellets were re-suspended with Dulbecco’s modified Eagle’s medium (DMEM) containing 10% fetal bovine serum and subjected to four cycles of freezing and thawing, followed by centrifugation at 2,400 x g for 10 min to remove cell debris. Supernatant- or cell-derived samples were then layered on top of 30 ml of a 10% to 50% Opti-prep density gradient medium (Sigma-Aldrich) prepared in DMEM and centrifuged in a Beckman SW41Ti rotor (Beckman) at 25,000 rpm for 16 hr at 4°C. Fractions of 1 ml were collected from the bottom of each tube.

### Confocal laser scanning microscopy analysis

Immunostaining of LD, NS5A and Core were performed as previously described [41]. Subcellular localization of HCV proteins was observed using an *Olympus* FLUOVIEW *FV1000* confocal laser scanning microscope. Co-localization of NS5A with Core or LD was quantified via Pearson’s correlation coefficient (PCC) [16] and intensity correlation quotient (ICQ) [17] using ImageJ software (http://imagej.nih.gov/ij/).

## Supporting Information

S1 Text Supplementary Materials and Methods. (DOCX)

S1 Fig Quantitative analysis of in vitro synthesized HCV RNA by 5’ UTR and NS5B-specific qRT-PCRs. (**A**) Schematic representation the qRT-PCR targets in HCV genome. The amplicons of two qRT-PCR assays together with their locations in the HCV genome were shown. (**B**) In vitro synthesized HCV RNA with approximate full genome (IVT-F RNA) was examined with Agilent RNA 6000 Nano Kit, in which 150 ng of RNA was used. (**C**) RNA copy numbers of IVT-F at each dilution were determined by two qRT-PCR assays with respective RNA standard. (**D**) The molecular ratios of NS5B to 5’ UTR of IVT-F in each dilution. The mean value of their ratios was set as the reference ratio. Values are presented as mean ± SEM, n = 2.
(TIF)

S2 Fig Characteristics of HCV RNAs in cell cultures. (**A**) Distribution of HCV RNA (J6/JFH-1) in fractions from culture supernatant. Detailed information was described in Fig 1. (**B**) The 3’:5’-end ratios of fractions in Fig 1B. Normalized 3’:5’-end ratios of density fractions from cultures infected with HCVcc (JFH-1). The ratio values calculated from NS5B (3’ end) and 5’ UTR (5’ end) qRT-PCRs were normalized by the reference ratio (0.459; S1D Fig).
(TIF)

S3 Fig Northern blotting examination of HCV RNAs. 10 μg of total RNA isolated from HCV infected or naïve Huh7.5.1 cells and 200 ng IVT-F RNA were loaded for detection.
(TIF)

S4 Fig Production of HCVtcp from replication-competent or replication-defective subgenomes (WT or GND, respectively), with benzonase treatment of the inoculum. Detailed information of production and detection of HCVtcp was described in Fig 2. Expression of the subgenomes and Core in the producer cells were shown in Fig 2B. The HCVtcp containing medium was treated with 30 IU/ml of benzonase for 1 hr before inoculating to naïve Huh7.5.1 cells. HCVtcp production was determined by quantification of the viral RNA in the transduced cells. Results shown represent the mean ± SEM, n = 4. HCV RNA copies are indicated as numbers per μg of total RNA.
(TIF)

S5 Fig Agarose gel electrophoresis of RNA fragments used in AlphaScreen assay. 1.5 μg of each RNA fragments were loaded to a 1% denaturing agarose gel for electrophoresis.
(TIF)

S6 Fig Production of HCVtcp packaged with EmGFP RNA. (**A**) Schematic representation of the plasmids used in a trans-complementation system for producing HCVtcp carrying EmGFP RNA. pCAG/NS3-5B expresses NS3, NS4A, NS4B, NS5A, and NS5B under the CAG promoter. p/EmGFP encodes EmGFP. p/EmGFP-J3UTR and pEm/GFP-H3UTR contain a 3’ UTR sequence derived from JFH-1 or H77c isolates, respectively, downstream of the stop codon of EmGFP. p/5’UTR-EmGFP contains the 5’ UTR sequence of JFH-1 upstream of the start codon of EmGFP. p/5’UTR-EmGFP-J3’UTR, encoding EmGFP flanked by 5’UTR and 3’UTR of JFH1. (**B**) Expression of EmGFP in producer cells with or without co-expression of NS3-5B. The scale bar indicates 200 μm. (**C**) Expression of NS5A and Core in producer cells. Identities of the transfected plasmids are indicated at the top, as referenced in Fig 4C. (**D**) Detection of production of HCVtcp packaged EmGFP-3’ UTR RNA, with pretreatment of the virions containing medium by 30 IU benzonase for 1 hr. N/G3J and cont were as described in Fig 3. The results shown represent the mean of three independent experiments ± SEM. EmGFP RNA copies are indicated as numbers per μg of total RNA. (**E**) Competition between RNA fragments with 3’UTR for interaction with Core. 4 nM of non-labeled 3’UTR, 5’UTR, or GFP were pre-incubated with 20 nM Core for 30min and then mixed with 4 nM biotinylated 3’UTR (Bio-3’UTR), followed by AlphaScreen assay. pc: the reaction with 4 nM of Bio-3’UTR; nc: the reaction without RNA fragments. Values are the means ± SEM, n = 2.
(TIF)

S7 Fig Steady-state levels of mutant subgenomic HCV RNAs. Plasmids expressing subgenomic HCV mutants (Fig 5A) were transfected into Huh7.5.1 cells, followed by total RNA extraction 48 hr post-transfection. The RNA was treated with Dnase to clear of plasmid DNA. cDNA was synthesized using a VILO superscription kit. A ~2.8 kb DNA fragment spanning the complete region of NS5A and part of NS5B (from nt 6234 to 9040) in the JFH-1 genome, was amplified with a forward primer, 5’-CACAATTGGATAACTGAGGACTGCCCCATCC-3’, and a reverse primer 5’- GTGCATAGAAAAGGCGTCAAGCCCGTG-3’. Two independent PCR sets with same parameters except amplification cycles (18 and 30 cycles, respectively) were carried out to catch the status of linear amplification. The relative intensities of each band were calculated with ImageJ software.
(TIF)

S8 Fig The effect of apical mutations in stem-loops I and II of 3’UTR on JFH-1 replication and packaging. LI&IIM Mutations in Fig 5E were introduced into full-length HCV genome in a pHH-based construct, pHH-JFH1 (WT), resulted in pHH-JFH1-X-LM (X-LM). Huh7.5.1 cells were transfected with these plasmids, followed by determination of RNA replication and virus production. HCV RNA in transfected cells were presented as relative abundance, which was determined by normalization of HCV RNA amount at 72 hr by the amount at 12 hr post-transfection (left panel). Virus production was determined by inoculation of naïve Huh7.5.1 cells with the culture supernatant at 72 hr post-transfection. HCV RNA in the infected cells was determined 48 hr post-infection (right panel). Values are presented as mean ± SEM, n = 4. HCV RNA copies are indicated as numbers per μg of total RNA.
(TIF)

S9 Fig Predicted structures of STIM and STIIM RNA fragments. The RNA structures were generated by predicting the lowest free energy structure and a set of low free energy structures for the sequences, by using an RNAstructure software. The dashed line circles marked the loop motifs existing in the loops of wild-type SLI and SLII (as shown in Fig 5C).
(TIF)
