# Supplementary material for: Involvement of the 3’ Untranslated Region in Encapsidation of the Hepatitis C Virus
Source: PLoS Pathog. 2016 Feb 11;12(2):e1005441. doi: 10.1371/journal.ppat.1005441 (PMC4750987; doi:10.1371/journal.ppat.1005441)
Supplement: S1 Text — (DOCX) [file ppat.1005441.s001.docx]

**Involvement of the 3’ Untranslated Region in Encapsidation of the Hepatitis C Virus Genome**

Guoli Shi,^1^ Tomomi Ando,^2,3^ Ryosuke Suzuki,^2^ Mami Matsuda,^2^ Kenji Nakashima,^1^ Masahiko Ito,^1^ Tsutomu Omatsu,^4^ Mami Oba,^4^ Hideharu Ochiai,^5^ Takanobu Kato,^2^ Tetsuya Mizutani,^4^ Tatsuya Sawasaki,^6^ Takaji Wakita,^2^ and Tetsuro Suzuki^1,*^

^1^ Department of Infectious Diseases, Hamamatsu University School of Medicine, Shizuoka, Japan, ^2^ Department of Virology II, National Institute of Infectious Diseases, Tokyo, Japan, ^3^ Division of Virology, Department of Microbiology and Immunology, Institute of Medical Science, University of Tokyo, Tokyo, Japan, ^4^ Research and Education center for Prevention of Global Infectious Diseases of Animals, Tokyo University of Agriculture and Technology, Tokyo, Japan, ^5^ Research Institute of Biosciences, Azabu University, Kanagawa, Japan, ^6^ Proteo-Science Center, Ehime University, Ehime 790-8577, Japan.

Competing interests: The authors have declared that no competing interests exist.

* E-mail: [tesuzuki@hama-med.ac.jp](mailto:tesuzuki@hama-med.ac.jp)

**Supplementary Materials and Methods**

**Plasmids**

Plasmids pHHJFH1, pHH/SGR-JFH1, and pHH/SGR-JFH1/GND, which express HCV genes under the Pol I transcription system, and pCAG/C-NS2, which expresses HCV Core-NS2 under the CAG promoter, have been previously described [[1](#_ENREF_1),[2](#_ENREF_2)]. pCAG/NS3-5B, encoding the nonstructural proteins NS3, NS4A, NS4B, NS5A, and NS5B in one open reading frame, was constructed as follows: a PCR fragment covering NS3 to NS5B of the HCV JFH-1 isolate was digested with EcoRI and XhoI, followed by insertion into the corresponding site of pCAG-Neo (Wako Pure Chemical Industry). The subgenomic replicon-based plasmids pHH/SGR-JFH1/Gluc and pHH/SGR-JFH1/Gluc/GND, which carry the *Gaussia* *princeps* luciferase (Gluc) gene as a reporter, were constructed using an infusion-PCR-cloning kit (Clontech Laboratories) with pHH/SGR-JFH1 and the replication-defective pHH/SGR-JFH1/GND as templates for PCR. Based on these two plasmids, plasmids encoding mutants of JFH-1 replicons (pHH/SGR-JFH1/Gluc/GND/∆5’UTR, ∆3’UTR, ∆3’X, ∆SLII&III, ∆SLI&II, ∆SLIII, ∆SLII, ∆SLI, LI &II M, STIM and STIIM) were constructed using PCR mutagenesis methods. To generate pBS/5’UTR, pBS/SL9038-SL9198, pBS/CRE3’UTR, pBS/CREVSL, pBS/CRE, and pBS/3’UTR for *in vitro* transcription, the corresponding fragments; the 5’ UTR, fragment covering nt 9038 to 9257, the terminal 163 nt of the 3' end of NS5B flanked by 3’ UTR, the terminal 163 nt of the 3' region of NS5B with the variable region and poly(U/UC) tract of the 3’ UTR, the terminal 163 nt of the 3' region of NS5B, and the 3’ UTR, were amplified by PCR, followed by EcoRI and BamHI digestion and ligated to pBluescript II SK (+). pBS/3’X, used for *in vitro* transcription of 3’ X tail fragment, was constructed by deletion of the variable region and poly U/UC stretch of 3’ UTR in pBS/3’UTR construct. To generate pEU/core/wt, for *in vitro* translation, 24-nt of flag-tag and 576-nt of the Core fragments were amplified by PCR using pCAG/C-NS2am/WT as a template, followed by SpeI and BamHI digestion and inserted into pEU-E01 (CellFree Sciences). To generate p/EmGFP, an EmGFP cDNA fragment was amplified by PCR with pcDNA6.2/N-EmGFP-DEST (**Life Technologies**) as a template, followed by KpnI and XhoI digestion and inserted into pCAG-Neo. To generate p/EmGFP-J3’UTR and p/EmGFP-H3’UTR, 3’ UTR fragments derived from HCV JFH-1 and H77c isolates were amplified by PCR using pJFH1 (GenBank accession number AB047639) and pCV-H77c (GenBank accession number AF011751) as templates, followed by EcoRI and BamHI digestion and inserted into p/EmGFP. p/5’UTR-EmGFP, p/5’UTR-EmGFP-3’UTR were constructed using an infusion-PCR-cloning kit (Clontech Laboratories), with KpnI linearized p/EmGFP and p/EmGFP-J3’UTR plasmids as vector backbones, respectively; and the insertion 5’ UTR fragments were amplified with pHHJFH1 as a template. To generate pRluc and the pRluc-3’UTR, the EmGFP fragment of p/EmGFP and the p/EmGFP-J 3’UTR were exchanged with a *renilla luciferase* cDNA fragment amplified by PCR using pGL4.70 (Promega) as a template, followed by KpnI and XhoI digestion. pHHJFH1-X-LM was constructed by swapping the corresponding sequences in pHH/SGR-JFH1/Gluc/GND/LI&IIM using EcoRV and SspI.

**Cell culture and DNA transfection**

The human hepatoma cell line Huh7.5.1 (a gift from Francis V. Chisari, the Scripps Research Institute) and Huh7-25 [3] was cultured in Dulbecco’s modified Eagle’s medium (DMEM) supplemented with 10% fetal bovine serum (FBS), penicillin and streptomycin.

Plasmid DNAs were transfected with TransIT-LT1 (Mirus Bio) according to the manufacturer’s recommended procedure. At 72 hr post transfection, cells were used to determine the expression of HCV Core and NS5A by Western blotting or EmGFP by fluorescence microscopy.

**HCVcc infectivity assays**

Infectivity of HCVcc from the culture supernatant or from the cells was determined as previously described [4-6]. At two days post-infection, HCV RNA copy numbers in the infected cells were determined by qRT-PCR.

**Gluc assay and Western blotting**

Culture supernatant (20 µl) collected from cells expressing plasmids carrying the Gluc reporter were used for determining Gluc activity by using a BioLux *Gaussia* Luciferase Assay Kit (New England Biolabs). Western blotting was performed as described previously [[1](#_ENREF_1)]. Mouse monoclonal antibodies against GAPDH (Sigma Aldrich) or HCV Core and an anti-NS5A rabbit polyclonal antibody [[4](#_ENREF_3)] were used as primary antibodies. Goat anti-mouse- and anti-rabbit HRP-labeled IgGs were used as secondary antibodies. Blots were developed using the ECL-Prime chemiluminescence kit (GE Healthcare).

**Northern blotting**

Total RNAs from naïve or J6/JFH-1-infected Huh7.5.1 cells were isolated with Tri-reagent (Sigma-Aldrich). Their isolated RNAs and IVT RNA were denatured with RNA loading buffer at 70 °C for 10 min and rapid cooled on ice, followed by analyzing with 1.5% denaturing agarose gel electrophoresis for northern blot hybridization with the DIG Northern starter kit (Roche) according to manufacturer’s instructions. DIG-labeled anti-sense RNA probe complementary to 5’ UTR (nt 1-340) was used to detect HCV RNA.

**RNAs and Core protein for AlphaScreen assay**

Biotin-labelled RNAs were synthesized using a MEGAscript T7 kit (Applied Biosystems) together with Biotin-14-CTP (Life Technologies). The molar ratio of CTP to Biotin-14-CTP in the reaction mix was 4:1. The synthesized RNAs were treated with TURBO DNase (Applied Biosystems) and cleaned up using a NucleoSpin RNA clean-up kit (TaKaRa Bio). The synthesized RNA fragments were examined in a 1% denaturing agarose gel electrophoresis. Refolding of the resulting RNAs was done as follow: RNA was denatured at 90 °C for 1 min in 20 mM Tris-HCl (pH 7.8) with 140 mM KCl, followed by incubation at 60 °C for 15 min, with subsequent cooling slowly to 30 °C over a 15 min period, after which MgCl_2_ was added to a final concentration of 2.5 mM. The RNA solution was maintained at 30 °C for 15 min and transferred to 0 °C. N-terminal FLAG-tagged Core was synthesized *in vitro* using the WEPRO1240 Expression kit (CellFree Sciences). In brief, firstly mRNA was synthesized with a pEU-E01 vector (a plasmid specifically designed for wheat-germ cell free protein synthesize system) containing the entire coding region of Core with FLAG tag sequence under the SP6 promoter. The resultant RNA was directly subjected to *in vitro* translation in a reaction containing WEPRO1240 wheat germ extracts, creatine kinase and SUB-AMIX and the mixture was incubated at 25 °C for overnight.

**S References**

1. Masaki T, Suzuki R, Saeed M, Mori K, Matsuda M, et al. (2010) Production of Infectious Hepatitis C Virus by Using RNA Polymerase I-Mediated Transcription. J Virol 84: 5824-5835.

2. Suzuki R, Saito K, Kato T, Shirakura M, Akazawa D, et al. (2012) Trans-complemented hepatitis C virus particles as a versatile tool for study of virus assembly and infection. Virology 432: 29-38.

3. Akazawa D, Date T, Morikawa K, Murayama A, Miyamoto M, et al. (2007) CD81 expression is important for the permissiveness of Huh7 cell clones for heterogeneous hepatitis C virus infection. J Virol 81: 5036-5045.

4. Masaki T, Suzuki R, Murakami K, Aizaki H, Ishii K, et al. (2008) Interaction of hepatitis c virus nonstructural protein 5A with core protein is critical for the production of infectious virus particles. J Virol 82: 7964-7976.

5. Lai CK, Saxena V, Tseng CH, Jeng KS, Kohara M, et al. (2014) Nonstructural Protein 5A Is Incorporated into Hepatitis C Virus Low-Density Particle through Interaction with Core Protein and Microtubules during Intracellular Transport. PLoS One 9: e99022.

6. Maillard P, Walic M, Meuleman P, Roohvand F, Huby T, et al. (2011) Lipoprotein lipase inhibits hepatitis C virus (HCV) infection by blocking virus cell entry. PLoS One 6: e26637.
