# Supplementary figures and images for: Involvement of the 3’ Untranslated Region in Encapsidation of the Hepatitis C Virus
Source: PLoS Pathog. 2016 Feb 11;12(2):e1005441. doi: 10.1371/journal.ppat.1005441 (PMC4750987; doi:10.1371/journal.ppat.1005441)

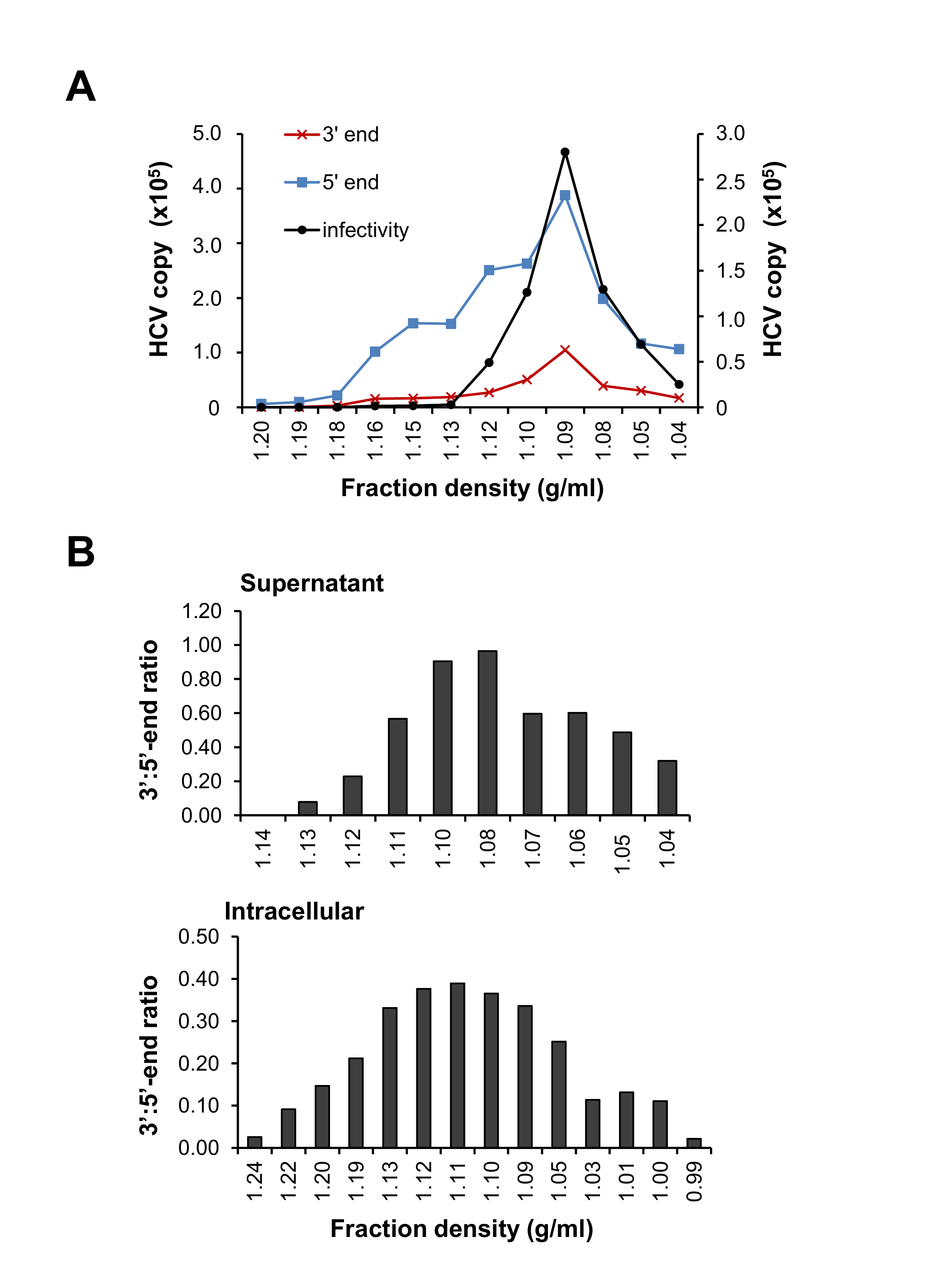

Supplement: S2 Fig — (A) Distribution of HCV RNA (J6/JFH-1) in fractions from culture supernatant. Detailed information was described in Fig 1. (B) The 3’:5’-end ratios of fractions in Fig 1B. Normalized 3’:5’-end ratios of density fractions from cultures infected with HCVcc (JFH-1). The ratio values calculated from NS5B (3’ end) and 5’ UTR (5’ end) qRT-PCRs were normalized by the reference ratio (0.459; S1D Fig). (TIF) [file ppat.1005441.s003.tif]

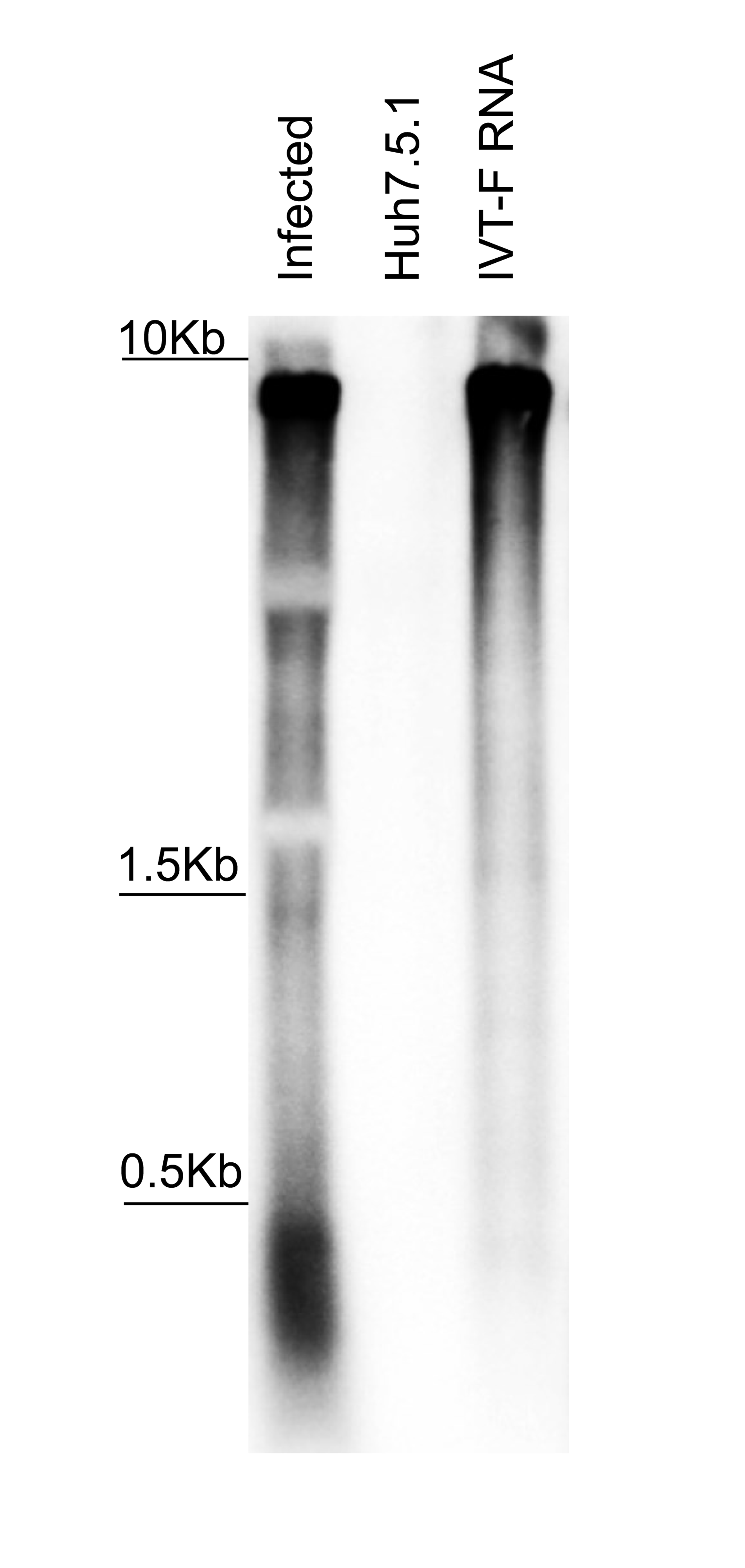

Supplement: S3 Fig — 10 μg of total RNA isolated from HCV infected or naïve Huh7.5.1 cells and 200 ng IVT-F RNA were loaded for detection. (TIF) [file ppat.1005441.s004.tif]

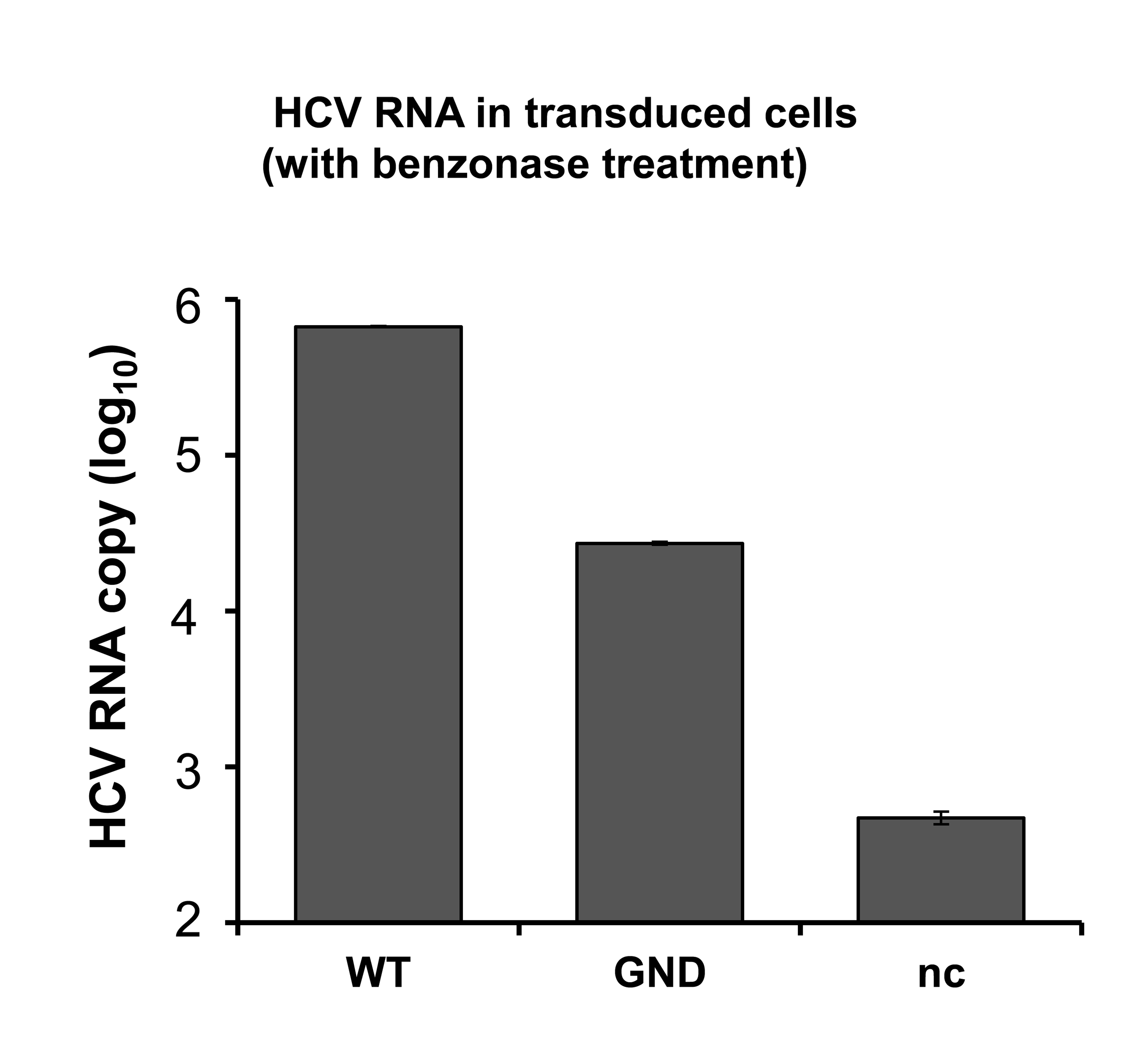

Supplement: S4 Fig — Detailed information of production and detection of HCVtcp was described in Fig 2. Expression of the subgenomes and Core in the producer cells were shown in Fig 2B. The HCVtcp containing medium was treated with 30 IU/ml of benzonase for 1 hr before inoculating to naïve Huh7.5.1 cells. HCVtcp production was determined by quantification of the viral RNA in the transduced cells. Results shown represent the mean ± SEM, n = 4. HCV RNA copies are indicated as numbers per μg of total RNA. (TIF) [file ppat.1005441.s005.tif]

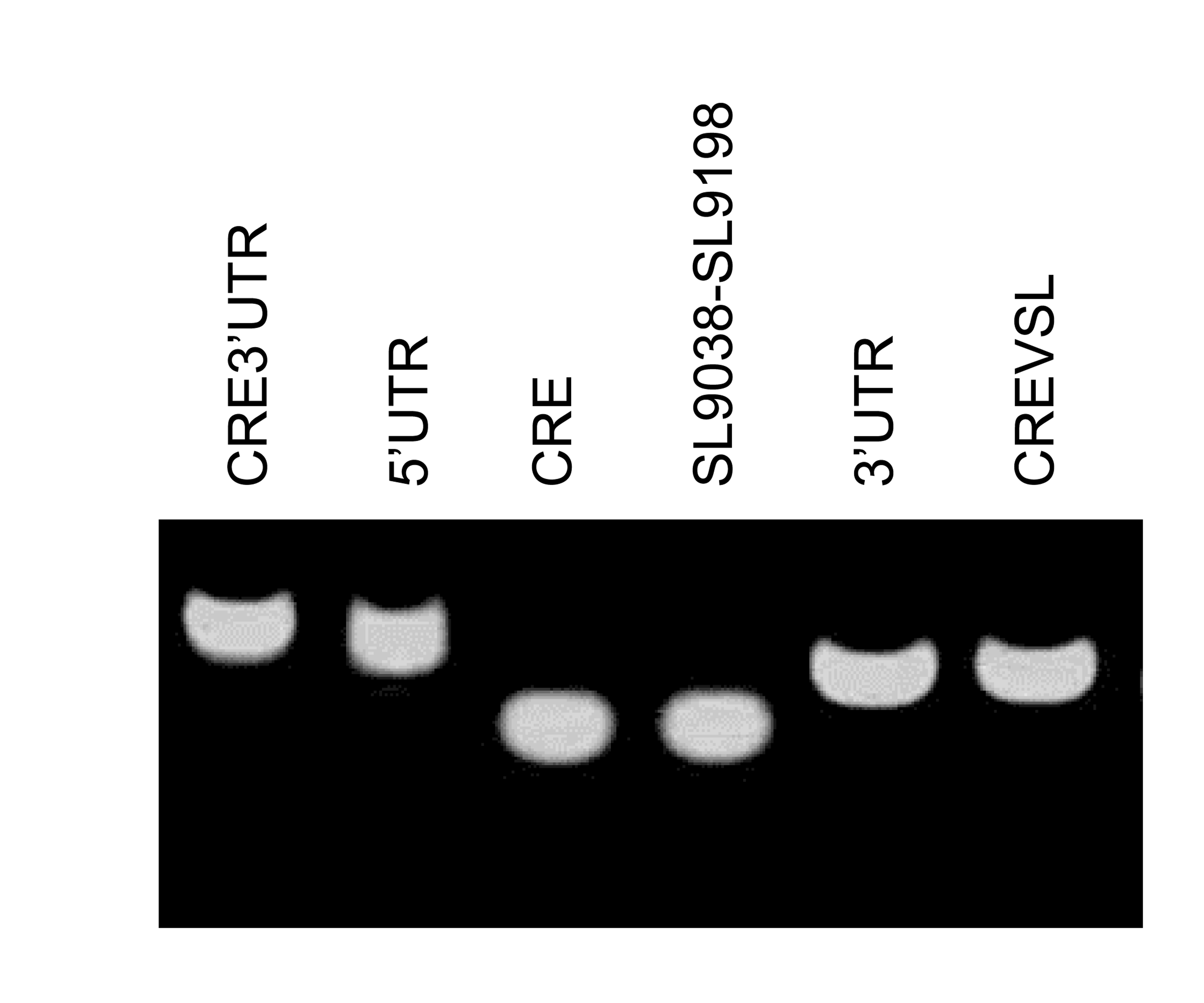

Supplement: S5 Fig — 1.5 μg of each RNA fragments were loaded to a 1% denaturing agarose gel for electrophoresis. (TIF) [file ppat.1005441.s006.tif]

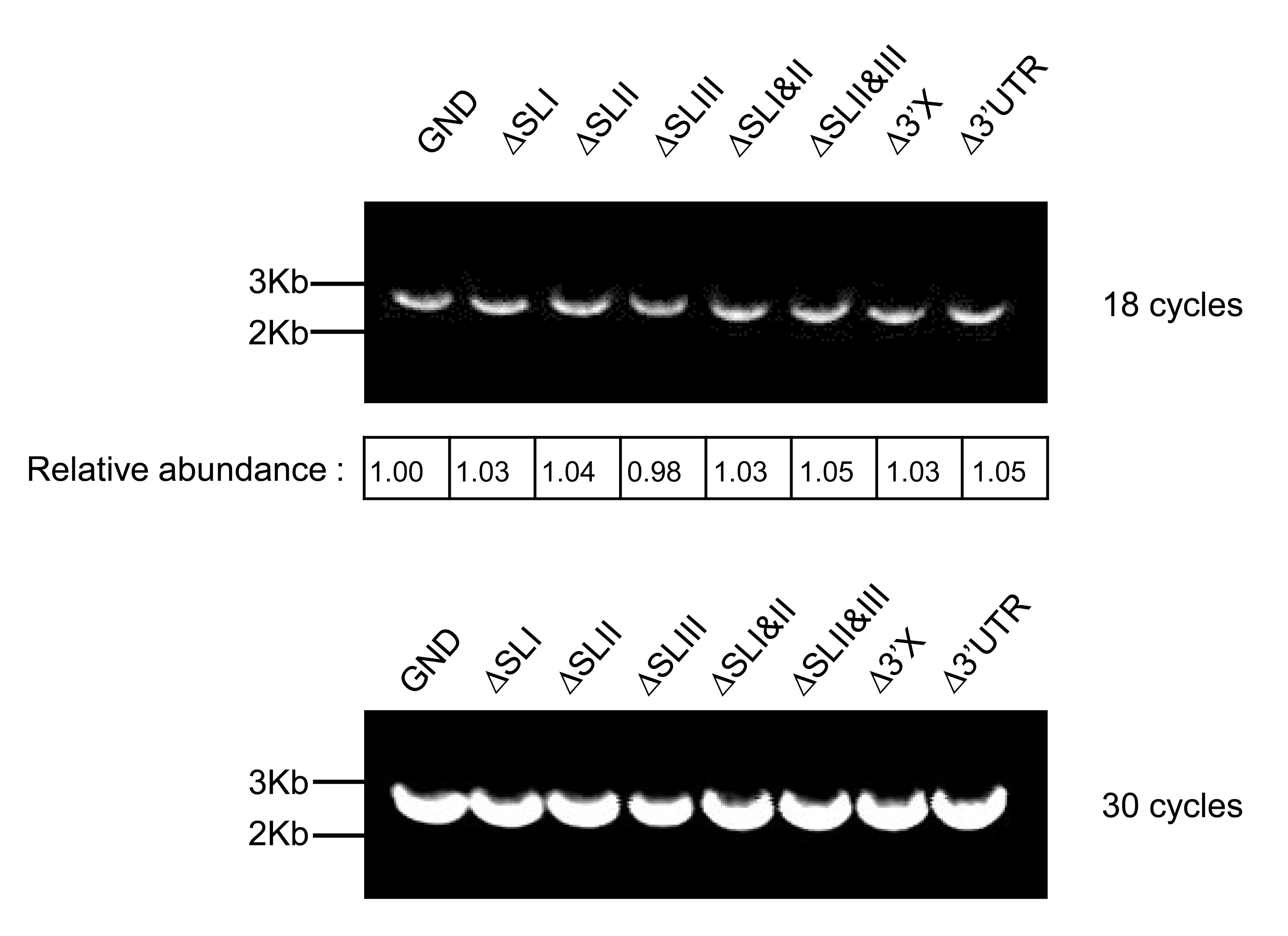

Supplement: S7 Fig — Plasmids expressing subgenomic HCV mutants (Fig 5A) were transfected into Huh7.5.1 cells, followed by total RNA extraction 48 hr post-transfection. The RNA was treated with Dnase to clear of plasmid DNA. cDNA was synthesized using a VILO superscription kit. A ~2.8 kb DNA fragment spanning the complete region of NS5A and part of NS5B (from nt 6234 to 9040) in the JFH-1 genome, was amplified with a forward primer, 5’-CACAATTGGATAACTGAGGACTGCCCCATCC-3’, and a reverse primer 5’- GTGCATAGAAAAGGCGTCAAGCCCGTG-3’. Two independent PCR sets with same parameters except amplification cycles (18 and 30 cycles, respectively) were carried out to catch the status of linear amplification. The relative intensities of each band were calculated with ImageJ software. (TIF) [file ppat.1005441.s008.tif]

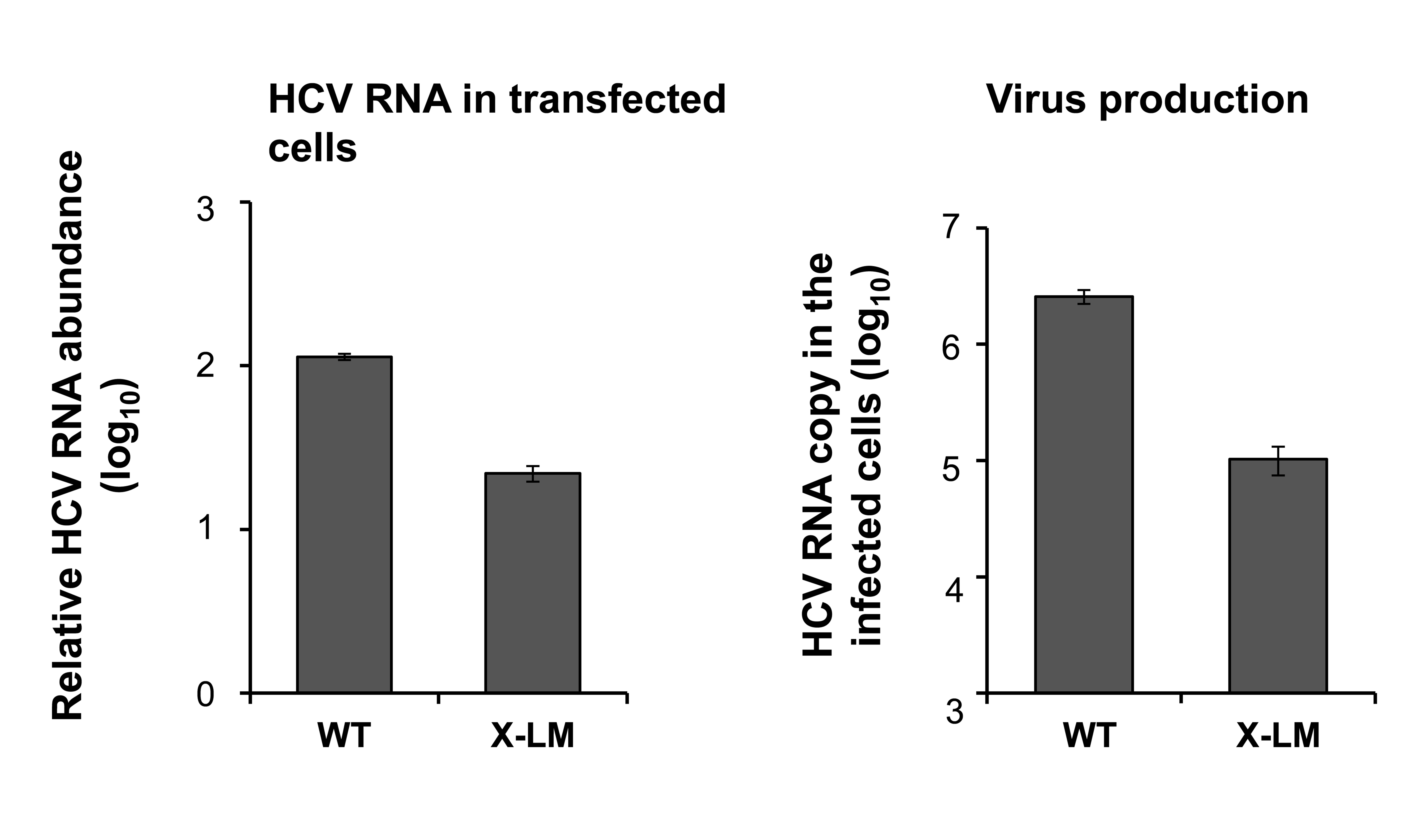

Supplement: S8 Fig — LI&IIM Mutations in Fig 5E were introduced into full-length HCV genome in a pHH-based construct, pHH-JFH1 (WT), resulted in pHH-JFH1-X-LM (X-LM). Huh7.5.1 cells were transfected with these plasmids, followed by determination of RNA replication and virus production. HCV RNA in transfected cells were presented as relative abundance, which was determined by normalization of HCV RNA amount at 72 hr by the amount at 12 hr post-transfection (left panel). Virus production was determined by inoculation of naïve Huh7.5.1 cells with the culture supernatant at 72 hr post-transfection. HCV RNA in the infected cells was determined 48 hr post-infection (right panel). Values are presented as mean ± SEM, n = 4. HCV RNA copies are indicated as numbers per μg of total RNA. (TIF) [file ppat.1005441.s009.tif]

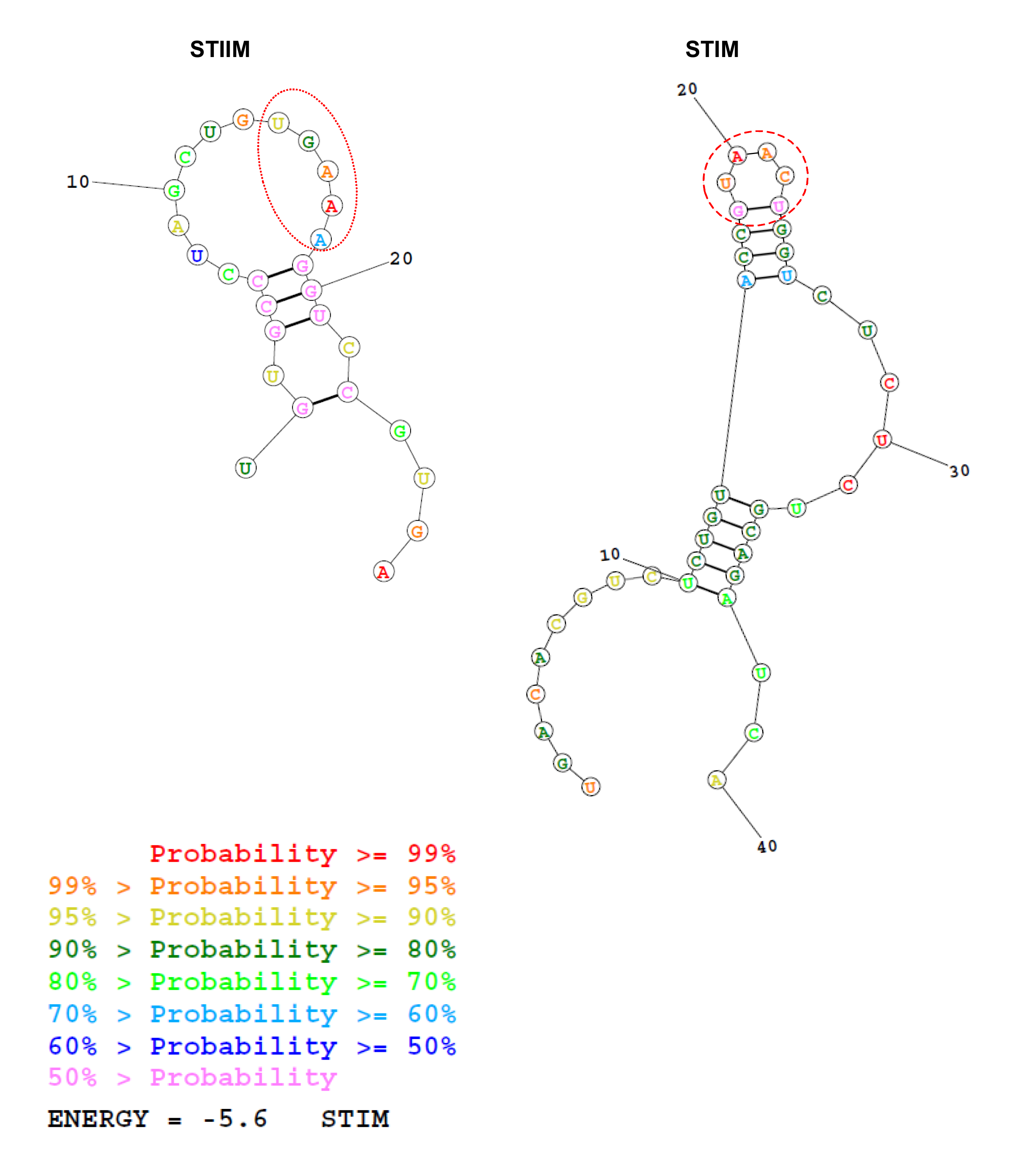

Supplement: S9 Fig — The RNA structures were generated by predicting the lowest free energy structure and a set of low free energy structures for the sequences, by using an RNAstructure software. The dashed line circles marked the loop motifs existing in the loops of wild-type SLI and SLII (as shown in Fig 5C). (TIF) [file ppat.1005441.s010.tif]
